# Identification of an active miniature inverted‐repeat transposable element *mJing* in rice

**DOI:** 10.1111/tpj.14260

**Published:** 2019-03-01

**Authors:** Yanyan Tang, Xin Ma, Shuangshuang Zhao, Wei Xue, Xu Zheng, Hongying Sun, Ping Gu, Zuofeng Zhu, Chuanqing Sun, Fengxia Liu, Lubin Tan

**Affiliations:** ^1^ State Key Laboratory of Plant Physiology and Biochemistry China Agricultural University Beijing 100193 China; ^2^ National Center for Evaluation of Agricultural Wild Plants (Rice) MOE Laboratory of Crop Heterosis and Utilization Department of Plant Genetics and Breeding China Agricultural University Beijing 100193 China

**Keywords:** amplification, DNA transposon, MITE, targeted high‐throughput sequencing, rice

## Abstract

Miniature inverted‐repeat transposable elements (MITEs) are structurally homogeneous non‐autonomous DNA transposons with high copy numbers that play important roles in genome evolution and diversification. Here, we analyzed the rice *high‐tillering dwarf* (*htd*) mutant in an advanced backcross population between cultivated and wild rice, and identified an active MITE named *miniature Jing* (*mJing*). The *mJing* element belongs to the *PIF*/*Harbinger* superfamily. *japonica* rice var. Nipponbare and *indica* var. 93‐11 harbor 72 and 79 *mJing* family members, respectively, have undergone multiple rounds of amplification bursts during the evolution of Asian cultivated rice (*Oryza sativa* L.). A heterologous transposition experiment in *Arabidopsis thaliana* indicated that the autonomous element *Jing* is likely to have provides the transposase needed for *mJing* mobilization. We identified 297 *mJing* insertion sites and their presence/absence polymorphism among 71 rice samples through targeted high‐throughput sequencing. The results showed that the copy number of *mJing* varies dramatically among Asian cultivated rice (*O. sativa*), its wild ancestor (*O. rufipogon*), and African cultivated rice (*O. glaberrima*) and that some *mJing* insertions are subject to directional selection. These findings suggest that the amplification and removal of *mJing* elements have played an important role in rice genome evolution and species diversification.

## Significance Statement

An active MITE in rice, named *mJing*, was identified by analyzing a spontaneous high‐tillering dwarf mutant in an advanced backcross population between cultivated and wild rice. Through targeted high‐throughput sequencing, we identified 297 *mJing* insertion sites in rice and found that the copy number of *mJing* varies dramatically among *O. sativa*,* O. rufipogon*, and *O. glaberrima*, suggesting that the amplification of *mJing* plays an important role in rice genome evolution.

## Introduction

Transposable elements (TEs) are major components of many plant and animal genomes and were once regarded as ‘selfish DNA’ or ‘junk DNA’ (Doolittle and Sapienza, 1980; Orgel and Crick, 1980). However, increasing evidence suggests that TEs have made enormous contributions to the evolution of genome structure and the regulation of gene function (Bennetzen *et al*., 2005; Feschotte, 2008; Rebollo *et al*., 2012; Wang *et al*., 2013; Bennetzen and Wang, 2014) and have played a major role in the events shaping the genome leading to speciation (Kazazian, 2004). Therefore, understanding the mechanism underlying the origin and amplification of TEs should provide valuable insights into genome evolution and diversification.

Transposable element mobility can lead to genetic diversity, an important source of genetic variation for evolution (Bennett *et al*., 2004; Xiao *et al*., 2008; Naito *et al*., 2009; Studer *et al*., 2011; Huang *et al*., 2018). TEs are associated with adaptation to temperate climates in *Drosophila* (González *et al*., 2010). Aging in social insects is related to TE activity (Elsner *et al*., 2018), and *Alu* insertions into exons are generally deleterious and therefore face strong purifying selection in diverse human populations (Witherspoon *et al*., 2013). Functional variations caused by TE insertions were selected during crop domestication. Insertion of the transposon *Hopscotch* enhances the expression of the maize (*Zea mays*) domestication gene *tb1*, leading to increased apical dominance in maize compared with its wild ancestor, teosinte (Studer *et al*., 2011). A CACTA‐like transposon within *ZmCCT10* and a Harbinger‐like transposon within *ZmCCT9* occurred sequentially after the initial domestication of maize and were strongly selected to facilitate the adaptation of maize to higher latitudes (Yang *et al*., 2013; Huang *et al*., 2018). A dramatic loss of TEs from the coding regions of genes occurred during rice domestication (Li *et al*., 2017). Notably, TE‐mediated epigenetic regulation in crops also contributes to phenotypic variation to enhance environmental adaptation and trait improvement (Song and Cao, 2017).

Miniature inverted‐repeat transposable elements (MITEs) are short non‐autonomous Class II elements (100–800 bp) derived from autonomous families via internal deletion (Bureau *et al*., 1996; Feschotte and Mouchès, 2000; Jiang *et al*., 2003). MITEs have been identified in a wide range of organisms, including grasses (Bureau and Wessler, 1992), fungi (Yeadon and Catcheside, 1995), insects (Tu, 1997), and humans (Smit, 1996; Smit and Riggs, 1996). MITEs are the most abundant TEs in the rice genome. The MITE insertion has generated numerous polymorphisms, which serve as an ongoing source of genetic variation (Huang *et al*., 2008). MITEs play important roles in gene regulation and genome evolution, as they preferentially associate with the regulatory regions of rice genes (Lu *et al*., 2012). MITEs embedded in the regulatory regions of genes can also alter their translation levels. A *stowaway*‐like MITE insertion in the 3′‐untranslated region of the agronomically important gene *Ghd2* directly represses its protein synthesis, affecting grain number, plant height, and heading date in rice (Shen *et al*., 2017). As a consequence, the effect of MITEs on allelic variation accelerates the evolutionary process.

Although MITEs are widely distributed in plant and animal genomes, to date only a few active MITEs have been identified. In rice, the first active MITE, named *miniature Ping* (*mPing*), was identified in a mutant with slender glumes, which harbors an insertion of this element in the *slender glume* (*slg*) mutant allele (Nakazaki *et al*., 2003); this element was also identified through genomic/computational analysis (Jiang *et al*., 2003; Kikuchi *et al*., 2003). *mPing* belongs to the *Tourist*‐like MITE superfamily and is a perfect deletion derivative of *Ping*, the autonomous partner responsible for the mobilization of *mPing* (Jiang *et al*., 2003). Transposition of *mPing* can be enhanced by the loss‐of‐function of the *Rice ubiquitin‐related modifier 1* (*Rurm1*) gene and in response to cold and salt stress leading to the upregulation of nearby genes (Naito *et al*., 2009; Tsukiyama *et al*., 2013). In addition, three active MITEs, *nDart*,* dTok,* and *nDaiZ*, all belonging to the *hAT* superfamily, were identified in rice through analysis of the mutability of genes for albinism, multiple pistils, and golden hulls and internodes, respectively (Fujino *et al*., 2005; Moon *et al*., 2006; Huang *et al*., 2009). Finally, *mGing*, a member of the *Gaijing* family, can be activated under stress conditions including γ‐ray irradiation and tissue culture (Dong *et al*., 2012). Therefore, identifying these types of active elements opens up opportunities for understanding the origin and amplification of MITEs.

Here, we identified an active MITE in rice, named *miniature Jing* (*mJing*), belonging to the *PIF*/*Harbinger* superfamily, as well as the putative autonomous element *Jing*, which is responsible for the mobilization of *mJing*. Targeted high‐throughput sequencing showed that the copy number of *mJing* varies dramatically among Asian cultivated rice (*O*. *sativa*), its wild ancestor (*O*. *rufipogon*), and African cultivated rice (*O*. *glaberrima*), suggesting that the mobilization of *mJing* elements has played an important role in rice genome evolution and species diversification.

## Results

### Identification of a spontaneous high‐tillering dwarf mutant in an advanced backcross population between cultivated and wild rice

**Figure 1:**
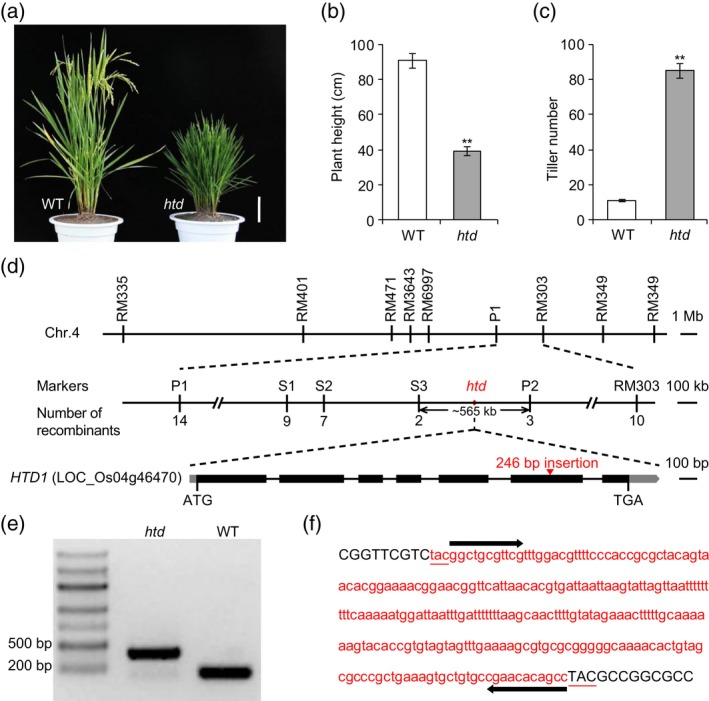
Identification of the miniature inverted‐repeat transposable element *miniature Jing* (*mJing*). (a) Phenotypes of the recipient parent Teqing (wild‐type, WT) and the *high‐tillering dwarf* (*htd*) mutant at the heading stage. Scale bars represemt 10 cm. (b) and (c) Comparison of plant height (b) and tiller number (c) between WT and *htd* plants at the harvest stage. Values are represented as mean ± SD (*n* = 20). Two‐tailed Student's *t*‐tests were performed between WT and *htd* (**P <* *0.01). (d) Mapping of *htd* and identification of the candidate mutant gene for the high‐tillering and dwarf phenotypes. The *htd* gene was mapped to a 565 kb interval on chromosome 4. The number of recombinant plants is shown below the corresponding markers. *HTD1* (LOC_Os04g46470) is a strong candidate gene due to the presence of a 246‐bp insertion in its coding region in the *htd* mutant compared with WT. Black boxes and black lines represent exons and introns, respectively. Gray boxes represent the 5′‐untranslated region (UTR) and 3′‐UTR. The red triangle represents the insertion site. (e) Validation of the insertion within *HTD1* gene by PCR analysis. (f) Sequences of the 246‐bp insertion and its flanking region. Red lowercase letters and black uppercase letters represent the *mJing* insertion and the 5′‐ and 3′‐flanking sequences of the *mJing* insertion in *HTD1*, respectively. Red underlining and black arrow indicate the target site duplications (TSDs) and terminal inverted repeats (TIRs) of *mJing* element, respectively.

A spontaneous mutant, referred to as *high‐tillering dwarf* (*htd*), was identified in an advanced backcross population (BC_3_) derived from a cross between a perennial wild rice (*Oryza rufipogon* Griff.) accession (YJCWR, Yuanjiang common wild rice) as the donor parent and *indica* variety Teqing as the recipient parent (also referred to as wild‐type, WT). The *htd* mutant has significantly reduced plant height, internode length, and grain number and significantly increased tiller number compared with WT (Figures 1a–c and S1). Moreover, *htd* has reduced grain width, grain thickness, and 1000‐grain weight compared with WT (Figure S1).

To identify the *HTD* gene, we developed a secondary F_2_ population (204 individuals) derived from a cross between the *htd* mutant and *indica* variety Teqing (WT). Phenotypic investigation revealed that the high‐tillering dwarf character is controlled by a single recessive gene (164 WT plants and 40 mutant plants; χ^2^ = 2.88 < χ^2^_0.05,1_ = 3.84). However, we did not detect polymorphic molecular markers linked to *HTD* on *O. rufipogon* introgression segments harbored by the mutant *htd* allele, suggesting that the causal mutation is present in the genome of the recipient parent. Therefore, we constructed another F_2_ population (738 individuals) derived from a cross between *htd* and *indica* variety 93‐11. Using 150 recessive homozygous plants, *htd* was delimited to a 565 kb interval between markers S3 and P2 on chromosome 4 (Figure 1d).

Based on annotation information for the rice reference genome (Os‐Nipponbare‐Reference‐IRGSP‐1.0, MSU7), we detected a gene with a known function within the fine‐mapping region of *htd*. This gene, *HIGH‐TILLERING DWARF1* (*HTD1*, LOC_Os04g46470), encodes carotenoid cleavage dioxygenase, which regulates axillary bud outgrowth (Zou *et al*., 2006). Hence, *HTD1* was a strong candidate for the *HTD* gene due to the similar phenotypic changes between the *htd1* and *htd* mutants. A sequence comparison of the *HTD1* coding region showed that the *htd* mutant had a 246‐bp insertion in exon 6 relative to the WT (Figure 1d,e), resulting in a premature stop codon and a new *htd1* mutated allele (Figure S2). These results suggest that the dysfunction of *HTD1* causes the high‐tillering dwarf phenotype of the *htd* mutant.

### Identification of *mJing*, an active MITE

Nucleotide sequence analysis showed that the 246‐bp insertion in *htd1* includes a 3‐bp target site duplication (TSD) of TAC and 11‐bp terminal inverted repeats (TIRs) of 5′‐GGCTGCGTTCG‐3′ (Figure 1f). BLAST analysis using Censor (http://www.girinst.org/, Kohany *et al*., 2006) revealed that the insertion fragment belongs to the *PIF*/*Harbinger* DNA transposon superfamily, suggesting that the insertion is a MITE. Additionally, its TIR sequence does not share high similarity with known MITEs, and therefore we designated the MITE identified in this study as *miniature Jing* (*mJing*).

**Figure 2:**
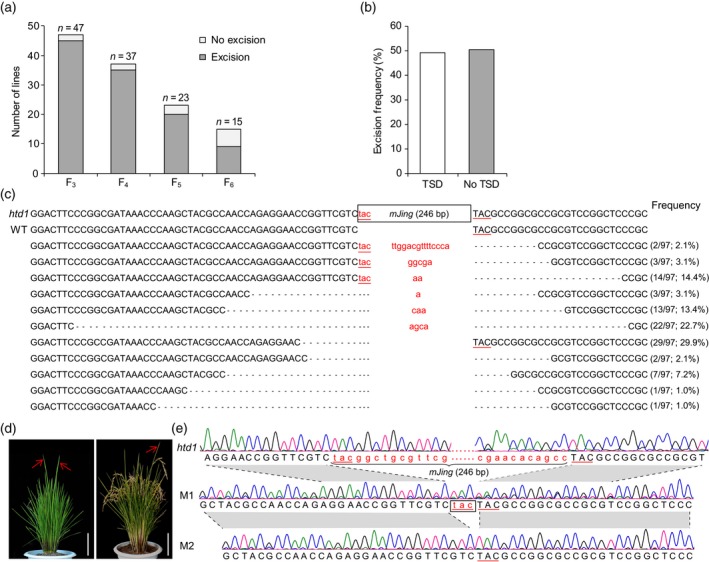
Excision of *mJing* within the *htd1* mutant allele. (a) Excision frequency of *mJing* within the *htd1* mutant allele in the F_3_ through F_6_ generations. The number of surveyed families in each generation is shown in the histogram. (b) Frequency comparison of excision with and without perfect TSDs for the 97 independent excision events. (c) Footprint analysis of the excision at the original site of *mJing*. The *mJing* insertion within the *htd1* mutant allele is shown in the box. Dashes represent the deleted bases after *mJing* excision. The numbers in parentheses indicate the proportion of corresponding excision events. (d) Phenotypes of chimeric mutants at the tillering stage (left) and the maturity stage (right). Red arrows show tillers with partial phenotypic recovery. Scale bar represemts 10 cm. (e) Two types of precise excision events at the original site of *mJing* in normal tillers of chimeric plants. Chromatograms show the sequences covering the original sites of *mJing* in dwarf tillers and taller tillers from chimeric plants. M1 and M2 are two normal tillers from different chimeric plants. Gray regions represent those sharing identical sequences. Back box represents the footprint left by *mJing* excision. In (c) and (e), red lowercase letters represent the *mJing* insertion, black uppercase letters represent the 5′‐ and 3′‐flanking sequences of the *mJing* insertion in *HTD1*, and red underlining indicates the target site duplications (TSDs) of *mJing* element.

We performed polymerase chain reaction (PCR) analysis using the primer set ID‐6 to assess the presence of the 246‐bp insertion in 955 F_4_ individuals with high‐tillering dwarf phenotype (15–33 F_4_ plants from the same F_3_ individual), which developed from 47 recessive F_3_ plants harboring the homozygous *mJing* insertion (*mJing* ^+^/*mJing* ^+^). All individuals from two F_3_ families had only a single 395‐bp PCR product, indicating that the *mJing* excision did not occur in these individuals (*mJing* ^+^/*mJing* ^+^). By contrast, among the 45 other F_3_ families, at least one individual per F_3_ family yielded PCR products of two different sizes (*mJing* ^+^/*mJing* ^−^) or a single PCR product similar to that of the WT (*mJing* ^−^/*mJing* ^−^) (Figure S3 and Table S1). These results suggest that the *mJing* excision at the *htd1* locus occurred in approximately 95.7% (45/47) of the F_3_ individuals. We further investigated the occurrence of the *mJing* excision in the F_4_ through F_6_ generations using the same approach and found that the frequency of *mJing* excisions was 94.6%, 87.0%, and 60.0% in the F_4_–F_6_ generations, respectively (Figure 2a and Table S1). These results indicate that *mJing* within the *htd1* gene is an active MITE and that the frequency of *mJing* excision gradually decreases as the generations advance.

To analyze the footprints generated by *mJing* excision, we identified 97 independent excision events in F_4_ individuals using PCR and sequenced the amplicons. Of the 97 excision events, 48 (approximately 49.5%) retained one of the duplicated TSDs (TAC) (Figure 2b). Among these, 19 (19.6%) of the 97 events maintained the 5′ TSD and 29 (29.9%) maintained the 3′ TSD (Figure 2c). Additionally, we detected 11 different footprints at the original site of *mJing* (Figure 2c). The *mJing* excision resulted in 6–45‐bp deletions at the 5′ and/or 3′ flanking region and/or 1–15‐bp insertions (Figure 2c). Although the locations of the breakpoints differed between excision events, the nucleotide at the 5′ donor site in all excision footprints was cytidine (C) and in most footprints, the nucleotide at the 3′ acceptor site was cytidine (C, 42/97) or guanine (G, 26/97). In addition, we analyzed the reduced amino acid sequences of the *htd1* alleles in which the *mJing* MITE was imprecisely excised, showing that the *mJing* excision caused several mutations, including the amino acid deletion, reading frame shift, and premature translation termination (Figure S4a). Therefore, the imprecise excision of *mJing* at the *htd1* locus was not able to rescue the function of *HTD1*, resulting in individuals with *mJing* ^+^/*mJing* ^−^ or *mJing* ^−^/*mJing* ^−^ genotypes that displayed a high‐tillering dwarf phenotype (Figure S4b).

### The *mJing* excision mainly occurred in somatic cells

We examined the excision at the original site of *mJing* and found that, among the 35 recessive F_3_ families (35/45, 77.8%), at least one F_4_ individual per family had a homozygous genotype without the *mJing* insertion (*mJing* ^−^/*mJing* ^−^). Further analysis of the footprints by sequencing the *htd1* amplicons showed that the excision patterns of homozygous individuals without the *mJing* insertion (*mJing* ^−^/*mJing* ^−^) were identical to those of heterozygous individuals (*mJing* ^+^/*mJing* ^−^) from the same F_3_ family, implying that the *mJing* excision detected in F_4_ individuals mainly occurred in somatic cells of the F_3_ generation.

Next, we observed the phenotypes of the families with homozygous *mJing* insertions (*mJing* ^+^/*mJing* ^+^) and found that several individuals had one or two tillers of normal height along with dwarf tillers, a characteristic of a chimeric mutant (Figure 2d). PCR analysis showed that the dwarf tillers contained a homozygous *mJing* insertion (*mJing* ^+^/*mJing* ^+^), whereas the taller tillers from the same mutant plants had a heterozygous *mJing* insertion (*mJing* ^+^/*mJing* ^−^). Sequencing of the footprints of *mJing* excision revealed that the *mJing* MITE within *htd1* was precisely excised in the taller tillers of the chimeric mutants and the entire *mJing* element and/or TAC duplication was removed leading to the production of normal tillers due to the rescuing of *HTD1* gene function (Figure 2e). Taken together, these results suggest that the *mJing* excision mainly occurred in somatic cells and that the chimeric phenotype for plant height was caused by the precise excision of *mJing* in somatic cells.

### Characteristics of the *mJing*‐like MITEs in the rice genome

**Figure 3:**
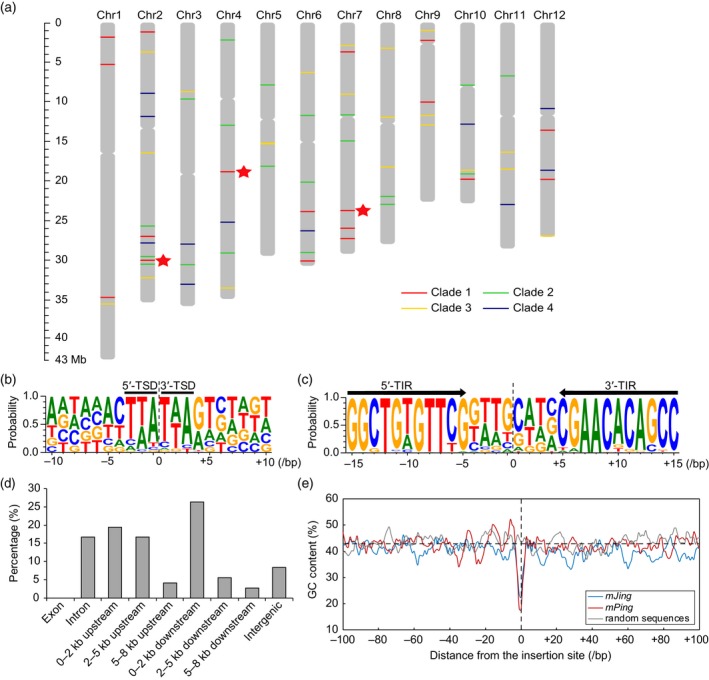
Characteristics of *mJing*‐like elements in the rice genome. (a) Distribution of the 72 *mJing*‐like elements in the *japonica* var. Nipponbare genome. Different‐colored dashes represent the location of each element belonging to four clades based on phylogenetic analysis of *mJing* family members, as shown in Figure 4. Red asterisks indicate the three *mJing*‐like elements with highly similar internal sequences to *mJing*. (b, c) Consensus sequences of target site duplications (TSDs) (b) and terminal inverted repeats (TIRs) (c) of the 72 *mJing*‐like elements in the Nipponbare genome. The size of each letter indicates the frequency of the corresponding nucleotide. Black lines and arrows above the letters indicate the TSDs and TIRs in (b) and (c), respectively. (d) Insertion preferences of the 72 *mJing*‐like elements in the Nipponbare genome. (e) Sliding‐window analysis of GC content within the flanking regions of *mJing*‐ and *mPing*‐like elements. Red and blue lines indicate the average GC content in the flanking sequences of *mJing*‐ and *mPing*‐like elements, respectively, and the gray line represents that of randomly selected sequences from the Nipponbare genome as a control. The zero on the *x*‐axis indicates the insertion sites of both *mJing*‐ and *mPing*‐like elements.

To explore the characteristics of the *mJing* family in the rice genome, we performed a BLASTN analysis to identify *mJing*‐like MITEs in the reference genome of *O. sativa* ssp. *japonica* var. Nipponbare (Os‐Nipponbare‐Reference‐IRGSP‐1.0, MSU7). We identified 72 *mJing*‐like MITEs harboring the entire TSD and TIR sequences, which are randomly distributed on the 12 rice chromosomes (Figure 3a and Table S2). Through recovery and alignment of the TSD and TIR sequences of the 72 *mJing*‐like MITEs, we found that approximately 67.36% of the TSDs were TAA/TTA trinucleotides, whereas 21.53% and 11.11% of the TSDs contained single base or two base changes compared with the TAA/TTA target site sequences, respectively (Figure 3b and Table S3). The 72 *mJing*‐like MITEs contained a 11‐bp conserved TIR with the sequence 5′‐GGCTGTGTTCG‐3′ (Figure 3c). In the *indica* var. 93‐11 reference genome, a member of the other subspecies of Asian cultivated rice, we identified 79 *mJing* family members and found that both the TSD and TIR sequences were similar to those of *japonica* var. Nipponbare (Figure S5 and Table S4).

Investigation of the locations of 72 *mJing*‐like MITE insertions in the Nipponbare reference genome showed that 12 insertion events (approximately 16.67%) were located in intron regions and 33 events (approximately 45.83%) occurred 2 kb upstream and downstream of gene‐coding regions, whereas no insertion events occurred in exon regions (Figure 3d). The previous study showed that approximately 52.0% of the *de novo mPing* insertions (133 of 256) were within 3 kb of a coding region (Naito *et al*., 2006), which is consistent with the characteristics of *mJing* insertion sites. The results suggested that the insertion of both *mJing* and *mPing* elements were preferentially in the flanking region of the gene. Sliding‐window analysis of GC content at the flanking regions of *mJing* insertions, including 100‐bp up‐ and downstream genome sequences, showed that the flanking regions close to the *mJing* insertion sites had lower GC contents than those randomly selected 200‐bp genome sequences; this is consistent with the characteristics of *mPing* family members in rice (Figure 3e) (Naito *et al*., 2006). Taken together, these results suggest that *mJing* is preferentially inserted into T/A‐rich regions in the rice genome, especially the 2 kb flanking regions of genes.

**Figure 4:**
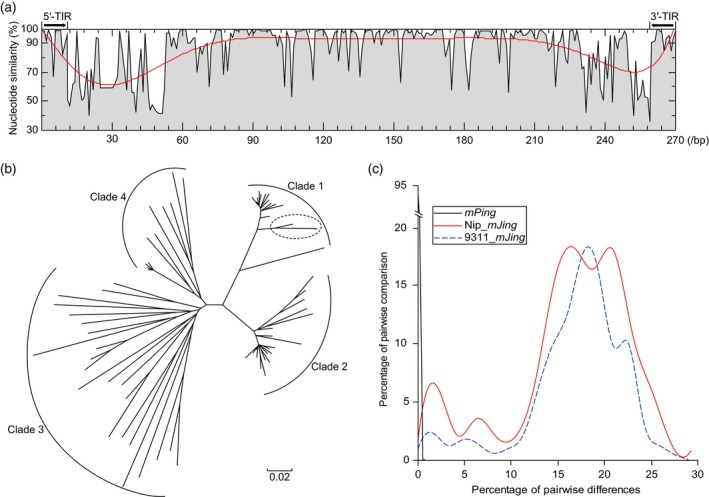
Sequence variation and amplification of *mJing*‐like elements in rice. (a) Analysis of sequence similarity among the 72 *mJing*‐like elements in the Nipponbare genome. Sequence similarity at each site was calculated based on sequence alignment via MUSCLE. Red curve represents the line of best fit for sequence similarity. (b) Phylogenetic analysis of the 72 *mJing*‐like elements in the Nipponbare genome. The black dotted oval represents the three family members (Nip_*mJing*2.10, Nip_*mJing*4.3, and Nip_*mJing*7.6) closest to *mJing*. (c) Frequency distribution of pairwise nucleotide diversity among *mJing* and *mPing* family members in rice, respectively. Black and red lines indicate *mPing*‐like and *mJing*‐like elements in the *japonica* var. Nipponbare genome, respectively. Blue dashed line indicates *mJing*‐like elements in the *indica* var. 93‐11 genome.

Sequence comparison of the 72 *mJing* family members revealed that, in addition to their conserved TSD and TIR sequences, *mJing* family members exhibit significant similarity among their internal sequences, whereas the sequences in the regions neighboring 5′ and 3′ TIRs are more polymorphic (Figure 4a). Phylogenetic analysis of the 72 *mJing* family members divided these MITEs into four clades (Figure 4b). Among these, Clade 1, comprising 18 *mJing*‐like MITEs, includes three members (Nip_*mJing*2.10, Nip_*mJing*4.3, and Nip_*mJing*7.6) closest to the *mJing* MITE identified in this study. To investigate the amplification of *mJing* family members in the rice genome, we calculated the pairwise nucleotide diversity among the 72 *mJing* TEs in *japonica* rice var. Nipponbare genome. The frequency distribution of pairwise nucleotide differences showed a four‐peak distribution that is consistent with the distribution characteristics of *mJing* family members in the *indica* var. 93‐11 genome (Figure 4c). By contrast, the *mPing* family, with 51 members in the Nipponbare genome, had substantially lower nucleotide diversity, and its frequency distribution of pairwise nucleotide differences showed a unimodal distribution (Figure 4c), pointing to different amplification patterns between the *mJing* and *mPing* families during rice evolution. Therefore, both phylogenetic and pairwise nucleotide difference analyses demonstrated that multiple rounds of *mJing* amplification have occurred during rice evolution, and the similar amplification patterns between the *indica* and *japonica* subspecies suggest that the amplification of the *mJing* family might have occurred prior to the *indica*−*japonica* differentiation.

### Identifying the putative autonomous element of *mJing*

The *mJing* MITE is a non‐autonomous element, implying that it has no capacity to encode an active transposase. Therefore, we reasoned that the transposition of *mJing* is catalyzed by a transposase encoded by its corresponding autonomous elements. To identify the corresponding autonomous elements of *mJing*, we used the *mJing* sequence to query the *indica* var. 93‐11 reference genomic with the MAK program (Yang and Hall, 2003a) and obtained more than 8000 high‐similarity sequences. We then selected 205 sequences that were >170 bp with an E‐value <10^−20^ and recovered the 10 kb upstream and downstream sequences surrounding the target sequences to manually identify the putative autonomous elements of *mJing*. We identified a putative autonomous element, referred to as *Jing*, located on chromosome 11 (19 319 940–19 323 561 bp) in the *indica* var. 93‐11 genome. BLASTN analysis revealed that three copies of the autonomous element *Jing* are present in the *japonica* var. Nipponbare genome, which are located on chromosome 2 (4 549 522–4 553 106 bp), chromosome 6 (22 387 549–22 391 278 bp), and chromosome 11 (22 941 196–22 944 833 bp).

**Figure 5:**
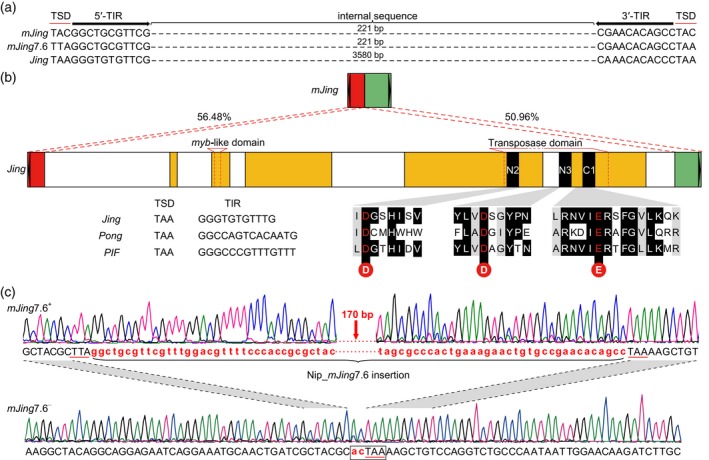
Identification of the putative autonomous element, *Jing*. (a) Sequence comparison of TSDs and TIRs among *mJing*, NIP_*mJing*7.6, and *Jing* elements. Red lines and black arrows above the bases represent TSDs and TIRs, respectively. Dashes represent the internal sequences of each element. (b) Comparison of *mJing* and *Jing* elements. Black triangles represent TIRs. Red and green boxes indicate the 5′ and 3′ regions with high sequence similarity between *mJing* and *Jing*, and the numbers show nucleotide identity. Yellow boxes represent exons, and N2, N3, and C1 represent putative catalytic domains. Nucleotide sequences of the TSDs and TIRs and alignment of conserved domains with the DDE motifs of rice *Jing*,* Pong,* and maize *PIF* elements are shown. (c) The detection of *mJing* excision in transgenic Arabidopsis plants carrying both *mJing*7.6 and *Jing* via Sanger sequencing. *mJing*7.6^−^ represents a transgenic plant in which the *mJing*7.6 element was excised, while *mJing*7.6^+^ represents a transgenic plant containing an entire *mJing*7.6 element. Black uppercase letters and red lowercase letters below the chromatogram represent the 5′‐ and 3′‐flanking and internal sequences of *mJing*7.6, respectively. Red lines above the bases represent TSDs. Black box represents the TSD of Nip‐*mJing*7.6 and its footprint after excision.

The autonomous element *Jing* is 3608‐bp long, including a 3‐bp TSD of TAA and an 11‐bp TIR (GGGTGTGTTTG) in the *indica* var. 93‐11 genome (Figure 5a). Although the sequence identity at the 5′ and 3′ sub‐terminal regions between *mJing* and *Jing* is 56.48 and 50.96%, respectively, both the TSD and TIR sequences of *Jing* are highly similar to those of *mJing* identified in this study, and six *mJing* TEs in the Nipponbare genome and seven in the 93‐11 genome shared identical TIR sequences with *Jing*. Therefore, we reasoned that *Jing* is most likely to be the autonomous partner of *mJing*. Gene annotation using FGENESH (http://linux1.softberry.com/berry.phtml) showed that *Jing* contains a 1803‐bp open reading frame encoding 600 amino acid residues with an *myb*‐like domain and a transposase domain (Figure 5b). Notably, the transposase domain also possesses a putative DDE motif (referred to as N2, N3, and C1) as its catalytic functional domain (Yuan and Wessler, 2011); this domain contains three highly conserved acidic amino acids (Asp−Asp−Glu) among *Jing* and *Pong* in rice and *PIF* in maize (Jiang *et al*., 2003) (Figure 5b).

To determine whether *Jing* drives the transposition of *mJing*, we developed two constructs for transformation in *Arabidopsis thaliana*. The construct p*35S*::*Jing* harbors a genome fragment including the entire open reading frame of *Jing* controlled by the *Cauliflower mosaic virus 35S* promoter (*CaMV 35S*), and the other construct p*mJing*7.6 carries a 2280‐bp genomic fragment surrounding *mJing* Nip_*mJing*7.6 from *japonica* var. Nipponbare, which is closest to *mJing* identified in this study. We simultaneously introduced both constructs into Arabidopsis and obtained 12 independent co‐transformed transgenic plants (Figure S6). PCR analysis showed that *mJing*7.6 was excised from one transgenic plant. Excision footprint analysis showed that, except for one 3′ TSD (TAA) and two nucleotides from the internal sequence, the *mJing* element was removed at the original site of *mJing*7.6 (Figure 5c). Taken together, our findings support the notion that the *Jing* element was likely to have provided the source of transposase for *mJing*.

### Amplification and selection of *mJing* in wild and cultivated rice

**Figure 6:**
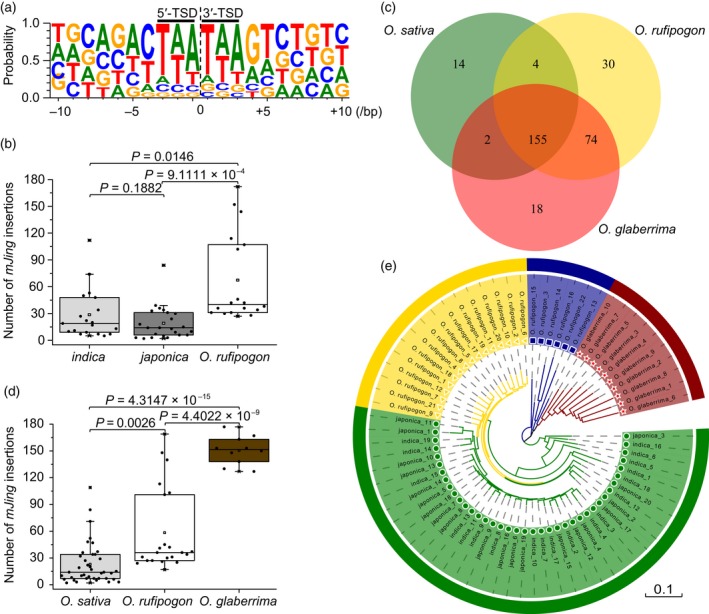
Amplification and selection of *mJing*‐like elements in wild and cultivated rice. (a) Consensus sequences of target site duplications (TSDs) of the 297 *mJing* elements in the rice genome. The letter size represents the frequency of the corresponding nucleotide. Black lines above the letters indicate TSDs. (b) Comparison of the number of *mJing* insertions among *indica*,* japonica,* and *O. rufipogon*. Black dots represent the number of *mJing* insertions in each sample. Two‐tailed Student's *t*‐tests were performed. (c) Venn diagrams showing the number of unique and common *mJing* insertions among *O*. *sativa*,* O*. *rufipogon,* and *O*. *glaberrima*. (d) Comparison of the number of *mJing* insertions among *O*. *sativa*,* O*. *rufipogon,* and *O*. *glaberrima*. Black dots represent the number of *mJing* insertions in each sample. Two‐tailed Student's *t*‐tests were performed. (e) Phylogenetic analysis based on the polymorphism of 297 *mJing*‐like elements in 71 accessions of wild and cultivated rice. Green represents Asian cultivated rice (*O. sativa*) varieties, including *indica* and *japonica* subspecies. Yellow and blue represent *O. rufipogon* accessions that are more and less closely related to cultivated rice, respectively. Red represents African cultivated rice (*O. glaberrima*) varieties.

To investigate the amplification and genome distribution of *mJing* in wild and cultivated rice, we conducted targeted high‐throughput sequencing to detect the *mJing* insertion sites in various cultivated and wild rice genomes. Because the target probes were designed based on the 5′ and 3′ sub‐terminal regions of *mJing*, which shared low similarity among family members, elements sharing highly similar sequences to *mJing* could be enriched. For example, in *japonica* var. Nipponbare, targeted high‐throughput sequencing detected three insertion events (Nip_*mJing*2.10, Nip_*mJing*4.3 and Nip_*mJing*7.6) with the highest sequence similarity to *mJing* among all *mJing* family members in the Nipponbare genome identified in this study. Through targeted high‐throughput sequencing, we identified 297 *mJing* insertion sites among all rice samples examined including 19 *indica* varieties, 20 *japonica* varieties, 22 *O. rufipogon* accessions, and 10 varieties of African cultivated rice (*O*. *glaberrima*) (Table S5); some *mJing*‐specific and/or unique insertions were verified by PCR (Figure S7). The 297 *mJing* insertions are distributed on the 12 rice chromosomes (Figure S8a), and the TSD sequences and insertion positions of the 297 *mJing* elements share similar characteristics with *mJing* family members in the Nipponbare reference genome (Figures 6a, S7b and Table S6). Interestingly, although approximately 47.81% (142/297) of the insertion events occurred in the 2 kb upstream and downstream sequences of gene‐coding regions, 6.06% (18/297) and 5.05% (15/297) insertions occurred in the intron and exon regions of functional genes, respectively (Figure S8b and Table S7), indicating that these *mJing* insertions might directly affect gene function.

Differences in the copy numbers of *mJing* between wild and cultivated rice might also reflect the amplification and selection of this MITE during rice evolution. We found that the copy number of *mJing* varied dramatically among the rice accessions examined, ranging from 2 to 177 members. The average copy number of *mJing* in the *O. rufipogon* genome was 58.3, which is significantly higher than that in *indica* (28.2) and *japonica* (18.1) rice (Figure 6b and Table S5). These results suggest that some *mJing* insertions with unfavorable genetic effects might have been removed during rice domestication. Additionally, although *O. sativa* shares 160 of 297 insertion sites with *O. rufipogon*, 104 and 16 unique insertion sites were detected in the *O. rufipogon* and *O. sativa* genomes (Figure 6c), respectively, suggesting that the *mJing* element maintained transpositional activation after the differentiation of wild and cultivated rice. Interestingly, the average copy number (150.6) of the *mJing* element in African cultivated rice (*O. glaberrima*) was dramatically higher than that in Asian cultivated (*O. sativa*) and wild rice (*O. rufipogon*) (Figure 6d), implying that the amplification of *mJing* might have played an important role in the evolution of Asian and African rice accessions with AA genomes.

Based on the polymorphisms (presence/absence) of 297 *mJing* MITEs (Table S5), our phylogenetic analysis divided the 71 rice samples into four divergent group, including one group of Asian cultivated rice, two groups of *O. rufipogon* accessions, and one African cultivated rice (*O. glaberrima*) group (Figure 6e). We calculated the fixation index (*F*_ST_) of each *mJing* locus between *indica* and *japonica*, between *O. sativa* and *O. rufipogon,* and between *O. rufipogon* and *O. glaberrima*. No obvious locus differentiation was detected between *indica* and *japonica* rice, whereas the *F*_ST_ values at 14 and 126 *mJing* loci were >0.25 between *O. sativa* and *O. rufipogon* and between *O. rufipogon* and *O. glaberrima* (Table 1). These results suggest that some *mJing* loci were directionally selected during rice evolution.

**Table 1:** Estimated fixation index (*F*_ST_) values of the 297 *mJing* insertions in rice

Population	*F*_ST_	Number of insertions	Percentage (%)
Between subspecies *japonica* and *indica*	<0.05	136	78.16
	0.05–0.15	34	19.54
	0.15–0.25	4	2.30
	>0.25	0	0
Between *O*. *rufipogon* and *O*. *sativa*	<0.05	165	59.35
	0.05–0.15	85	30.57
	0.15–0.25	14	5.04
	>0.25	14	5.04
Between *O*. *rufipogon* and *O*. *glaberrima*	<0.05	109	38.79
	0.05–0.15	24	8.54
	0.15–0.25	22	7.83
	>0.25	126	44.84

## Discussion

MITEs are widespread among eukaryotic genomes and play important roles in genome evolution and gene regulation. Previous studies have revealed the mechanisms underlying the emergence, spread, and disappearance of MITEs through the identification and characterization of active MITEs. Here, we identified an active MITE, *mJing*, by analyzing a spontaneous high‐tillering dwarf mutant obtained from an advanced backcross population from wild and cultivated rice. We also detected the *de novo* insertion sites of *mJing* in 14 F_5_ individuals, in which the *mJing* element was excised at the *htd1* locus, through the targeted high‐throughput sequencing. We found nine *de novo* insertion sites in the F_5_ individuals, indicating that a majority of elements excised may move to another site in the rice genome (Table S8). Additionally, both the TIRs and internal sequences of *mJing* are dramatically different from those of *mPing*, a previously identified active MITE in the rice genome, although the *mJing* and *mPing* elements share similar TSD sequences and belong to the *PIF/Harbinger* superfamily. Therefore, the identification of the *mJing* element provides opportunities to investigate the contribution of MITEs to the evolution of the rice genome.

TE activity is enhanced by biotic and abiotic stresses such as pathogen infection, γ‐irradiation, hydrostatic pressurization, and tissue culture (Orgel and Crick, 1980; Nakazaki *et al*., 2003; Xu *et al*., 2004; Lin *et al*., 2006). The dramatic difference in copy number of *mPing* between the two subspecies of rice, *japonica* and *indica*, suggests that, during rice domestication, extreme environmental conditions might have promoted the mobilization of *mPing* (Jiang *et al*., 2003). Indeed, the mobilization of *mPing* was detected in recombinant inbred lines derived from introgressive hybridization between *O. sativa* and *Zizania latifolia* (Shan *et al*., 2005). In addition to identifying an active MITE in the hybrid progenies of wild and cultivated rice, we performed targeted high‐throughput sequencing to analyze the *mJing* insertion in 10 wild rice introgression lines (Ma *et al*., 2016). We detected two *de novo* insertions (Table S9), suggesting that the mobilization of *mJing* might be induced by distance hybridization between wild (*O. rufipogon*) and cultivated (*O. sativa*) rice. This finding provides evidence to support the hypothesis that TEs are activated by ‘genomic shock’ due to plant‐wide hybridization (McClintock, 1984). Notably, the previous study demonstrated that *mPing* might have stimulated the loss‐of‐function of the *Rurm1* gene, supporting one of the models of genome shock theory that the endogenous stress enhances the transposition activity of TEs (Tsukiyama *et al*., 2013).

Because MITEs are non‐autonomous DNA elements that do not encode transposases, the mobilization of MITEs relies on the presence of transposase encoded by their cognate autonomous elements. In plants, most MITEs were likely to have derived from two DNA element superfamilies, *PIF/Harbinger* and *Tc1/Mariner* elements, which are classified as either *Tourist*‐like or *Stowaway*‐like elements (Feschotte *et al*., 2002). The *Tourist*‐like MITE *mPing* is a perfect deletion derivative of *Ping*, which belongs to the *PIF/Harbinger* superfamily and provides the source of transposase for *mPing* (Jiang *et al*., 2003). Unlike *mPing*, although *Stowaway*‐like MITEs are not deletion derivatives of *Tc1/Mariner* elements, the transposition of *Stowaway* MITEs is catalyzed by transposases encoded by *Tc1/Mariner* elements, demonstrating that transposases from distantly related elements play an important role in the movement of non‐deletion derivative MITEs (Feschotte *et al*., 2003; Yang *et al*., 2009). In addition, many MITEs are likely to have originated from members of the *En*/*Spm* and *Mutator* superfamilies, as evidenced by their shared TIR and TSD sequences (Wicker *et al*., 2003; Yang and Hall, 2003a,b).

Based on the current results, we hypothesize that, in rice, the *Jing* element is the source of the transposase for *mJing*, as revealed by the strong similarity of the TSD and TIR sequences between *Jing* and *mJing*. Furthermore, a heterologous transposition experiment showed that *mJing* can be excised by the transposase encoded by *Jing*. However, the sequence similarity in the sub‐terminal regions of *mJing* and *Jing* is lower than that between *mPing* and *Ping*. Therefore, we hypothesize that *mJing* might not be a deletion derivative of the existing autonomous element *Jing* but instead, the *mJing* element is likely to be mobilized by ‘borrowing’ a transposase from *Jing*, a distantly related autonomous element. Another hypothesis is that *mJing* is a deletion derivative of *Jing*, but the sequences in the sub‐terminal regions of *mJing* might have been altered during multiple rounds of amplification. Therefore, further investigations of the autonomous partner of *mJing* in rice or other plants including its evolutionary process would be valuable for understanding the mechanisms underlying the emergence, spread, and disappearance of MITEs.

The nucleotide diversity between elements from the same MITE family might reflect the amplification characteristics of MITEs during genome evolution. A study of the pairwise nucleotide diversity of full‐length elements from 37 MITE families in rice revealed the occurrence of different patterns of amplification bursts during rice evolutionary history (Lu *et al*., 2012). The *japonica* var. Nipponbare reference genome contains 51 members of the *mPing* family with high nucleotide similarity, implying that this family has undergone one round of rapid amplification. By contrast, the *mJing* family has 72 members with low nucleotide similarity in their 5′ and 3′ sub‐terminal regions, suggesting that this family experienced multiple rounds of amplification during rice evolutionary history. In addition, the average pairwise nucleotide diversity is higher for *mJing* versus *mPing* elements indicating that the amplification of *mJing* elements occurred before *mPing* amplification during rice genome evolution. Altogether, these results suggest that single or multiple rounds of amplification of these MITE families might have occurred during different periods of rice evolution.

Targeted high‐throughput sequencing is an efficient method for identifying the insertion positions of specific transposon elements in the genome. In the present study, we identified 297 *mJing* insertions in 71 rice samples through targeted high‐throughput sequencing. Notably, because the genome fragments were enriched via DNA hybridization, the insertions that were identified by targeted high‐throughput sequencing share highly sequence similarity with the *mJing* insertion in *HTD1*. In *japonica* var. Nipponbare, only three *mJing* family members with high sequence similarity to *mJing* in *HTD1* were detected, while in *O. rufipogon* accession YJCWR, 169 *mJing* family members were detected indicating that the copy number of *mJing* varies dramatically in cultivated versus wild rice. Subsequently, we found that the average copy number of *mJing* significantly differed between Asian cultivated rice (*O. sativa*) and its wild ancestor *O. rufipogon* and between *O. rufipogon* and African cultivated rice (*O. glaberrima*). African cultivated rice had the highest copy number (average of 150.6), while Asian cultivated rice had the lowest (average of 23.2) among the three species surveyed. In addition, 14 and 126 *mJing* loci might have undergone directional selection during the differentiation between *O. sativa* and *O. rufipogon* and between *O. rufipogon* and *O. glaberrima*, respectively. However, no obvious locus differentiation was detected between the two subspecies of Asian cultivated rice, *indica* and *japonica*. Whether environmental adaptation or purifying selection is associated with the variation in *mJing* copy number in the divergent rice genomes requires further investigation. Therefore, our identification of an active MITE *mJing* provides opportunities for investigating the roles of MITEs during rice evolution.

## Experimental procedures

### Plant materials

The *high‐tillering dwarf* (*htd*) mutant was identified from an advanced backcross population (BC_3_) derived from a cross between *O. rufipogon* accession YJCWR from Yuanjiang county, Yunnan Province, China, as the donor parent and *indica* rice variety Teqing (*O*. *sativa* ssp. *indica*) as the recipient parent (Tan *et al*., 2008). To map the gene for the high‐tillering, dwarf phenotype, an F_2_ population was developed from a cross between *indica* variety 93‐11 and the *htd* mutant. To investigate the amplification and genome distribution of *mJing*, 71 rice samples including 19 *indica* varieties, 20 *japonica* varieties, 22 *O. rufipogon* accessions, and 10 varieties of African cultivated rice (*O. glaberrima*) were used for targeted high‐throughput sequencing (Table S5).

### Phenotypic evaluation

Twenty plants each of the *htd1* mutant and recipient parent Teqing (WT) were examined to measure plant height, tiller number, internode length, grain length, grain width, and grain thickness.

### Footprint analysis of *mJing* excision within *HTD1*

Genomic DNA was extracted from 97 independent lines from the F_4_ populations and used for PCR analysis of *mJing* within the *HTD1* gene. Primers 6ID‐F and 6ID‐R were used to detect the insertion and excision of *mJing*. One homozygous (*mJing* ^−^/*mJing* ^−^) or heterozygous (*mJing* ^+^/*mJing* ^−^) individual per line was selected to analyze the *mJing* footprint based on analysis of the PCR products. The excision of *mJing* was confirmed by PCR amplification using the primer set Osmax6, and the PCR products were directly sequenced.

### Identification of an *mJing*‐like element in the rice reference genome

Both the *japonica* var. Nipponbare (Os‐Nipponbare‐Reference‐IRGSP‐1.0, MSU7) and *indica* var. 93‐11 (ASM465v1) reference genome sequences were downloaded from the Ensembl site (http://plants.ensembl.org/info/website/ftp/index.html). A local BLAST search was performed using the 243‐bp *mJing* element as a query with the following settings: length >120 bp and E‐value <10^−27^. If the length of the target sequence was <220 bp, its upstream and downstream sequences were extended until the 5′‐ and 3′‐TIRs appeared in the reference genome. In total, 72 and 79 full‐length *mJing*‐like elements were discovered in Nipponbare and 93‐11, respectively. Information about all of the *mJing*‐like elements is provided in Tables [Link], [Link], [Link].

### Characterization of *mJing*‐like elements in the rice reference genome

The consensus TSD and TIR sequences of *mJing*‐like elements in the rice genome were depicted using WebLogo 3.0 (Crooks *et al*., 2004). To analyze the insertion preference of *mJing*, the 100‐bp upstream and downstream sequences of the 72 *mJing*‐like and 51 *mPing*‐like insertion sites (including TSD sequences), respectively, were extracted from the Nipponbare genome. Furthermore, 72 200‐bp long sites were randomly selected from the Nipponbare genome as a control. The GC content was calculated for each 5‐bp sliding‐window at a 1‐bp increment. To investigate the association of *mJing*‐like elements and genes in the rice genome, the locations of *mJing* insertions were investigated, and based on the distance between the insertion site and the annotated genes, the insertion position was categorized as being in an exon or intron, upstream or downstream of a gene‐coding region, or in intergenic regions (>8 kb from a gene). Pairwise diversity among members of the *mPing* and *mJing* families in the Nipponbare genome was calculated using a Perl script (each gap was considered to be a single mismatch), and the frequency distribution of pairwise nucleotide diversity was used to describe the amplification event during genome evolution.

### Transposition assay

A 2280‐bp genomic fragment harboring the entire Nip_*mJing* 7.6 sequence with the 986‐bp 5′‐flanking region and 1051‐bp 3′‐flanking region was obtained through PCR amplification and inserted into the pCAMBIA1300 binary vector to generate the construct p*mJing*7.6. The p3*5S*::*Jing* construct harbored a genome fragment containing the entire gene‐coding region (1803 bp from ATG to TAG) of *Jing* controlled by the *Cauliflower mosaic virus 35S* promoter (*CaMV 35S*). Both constructs were introduced into *Agrobacterium tumefaciens* strain GV3103, followed by co‐transformation into *Arabidopsis thaliana* (Columbia ecotype). Transgenic plants were selected on Murashige and Skoog (MS) solid medium (0.7% (w/v) Phytagel) containing 30 mg L^−1^ hygromycin B. PCR analysis was performed using primer sets D_p*mJing*7.6‐2 and D_p*35S*::*Jing* to detect positive co‐transformed transgenic plants, respectively, and the amplicons were sequenced to analyze the footprint of the *mJing*7.6 excision.

### Targeted high‐throughput sequencing

Illumina sequencing libraries were constructed for targeted high‐throughput sequencing as described by Williams‐Carrier *et al*. (2010), with minor modifications. Genomic DNA was extracted using the cetyltrimethylammonium bromide (CTAB) method and treated with ribonuclease A (37°C) and proteinase K (42°C) for 1 h. Genomic DNA was sheared into approximately 400–500‐bp fragments by sonication (set to 15 sec on and 45 sec off and sonicated for 10 min). Modified adapters (Table S10) were ligated to the DNA fragments. DNA fragments containing *mJing* were enriched by hybridization to two biotinylated 40‐bp oligonucleotides of *mJing*, including the 11‐bp 5′‐TIR and its neighboring 29‐bp sub‐terminal sequence and the 11‐bp 3′‐TIR and its neighboring 29‐bp sub‐terminal sequence (Table S10). During PCR amplification to ‘bulk up’ the DNA, a 6‐bp index barcode in the middle of the P7 primers (index1–index64) was used to distinguish the samples (Table S10). Finally, the paired‐end libraries were sequenced on the Illumina HiSeq/MiSeq system at Novogene Company (Beijing, China). Approximately 0.1–0.5 G clean data were obtained per sample. The detailed method used for targeted high‐throughput sequencing is depicted in Figure S9.

### Sequencing data analysis

Raw reads were processed using Cutadapt (Martin, 2011) for adapter trimming, and quality filtered reads were aligned to the modified Nipponbare sequence (Os‐Nipponbare‐Reference‐IRGSP‐1.0, MSU7), excluding three sequences that are highly similar to *mJing* (Nip_*mJing*2.10, Nip_*mJing*4.3 and Nip_*mJing*7.6), using Bowtie2 (Langmead and Salzberg, 2012). The PTEMD program (Ye *et al*., 2009; Kang *et al*., 2016) was used to identify *mJing* insertions in the cultivated and wild rice genomes. The output format of PTEMD contained the insertion sites, TSDs, and breakpoints of each *mJing* insertion (Table S11). The genotypes of all samples were scored based on presence/absence polymorphisms of the *mJing* insertions. The fixation index (*F*_*ST*_) of each locus was calculated using DnaSP version 5.1 (Librado and Rozas, 2009).

### PCR validation of *mJing de novo* insertions

To validate the specific and/or unique *mJing* insertions in the cultivated and wild rice genomes, PCR primers were designed based on the flanking sequences of the *mJing* insertion, and locus‐specific PCR of the *mJing* insertion sites was performed. The products were separated using polyacrylamide gel electrophoresis (PAGE) in 1% (w/v) agarose gels to distinguish the presence/absence polymorphisms of *mJing* insertions in the rice genome. The primer sets used to detect the polymorphisms of the *mJing* insertions are listed in Table S12.

### Phylogenetic analysis

The sequences of 72 *mJing*‐like MITEs in the Nipponbare genome were aligned using MUSCLE (Edgar, 2004), and the phylogenetic tree was constructed using MEGA 6.0 and the neighbor‐joining method (Tamura *et al*., 2013). Based on the presence/absence polymorphisms of 297 *mJing* insertions, a phylogenetic tree of cultivated and wild rice was constructed using MEGA 6.0 (Tamura *et al*., 2013) and Evolview (He *et al*., 2016).

### Primers

The primers used in this study are listed in Table S12.

### Statistical analysis

Two‐tailed Student's *t*‐tests were performed using SPSS version 17 (SPSS Inc., Chicago, IL, USA). Statistical significance was set at *P *<* *0.05 and *P *<* *0.01.

## Conflict of Interest

The authors declare no conflict of interests.

## Author Contributions

LT and FL conceived and designed the work. YT performed most of the experiments. YT, XM, and XZ completed the bioinformatics analyses of all data. SZ, WX, HS, PG, ZZ, and CQ contributed to rice materials. YT, FL, and LT wrote the paper.

## Supporting information


**Figure S1.** Phenotypes of wild‐type (WT) and *htd* mutant rice.

**Figure S2.** Open reading frame (ORF) of *HTD1* and deduced amino acid sequences in WT and *htd*.

**Figure S3.** Transposition of *mJing* in a high‐tillering dwarf population.

**Figure S4.** Changes in amino acid sequence encoded by the *htd1* alleles and phenotypes of F_4_ individuals that have the *mJing* ^+^/*mJing* ^−^ or *mJing* ^−^/*mJing* ^−^ genotype.

**Figure S5.** Consensus sequences of target site duplications (TSDs) and terminal inverted repeats (TIRs) of 79 *mJing*‐like elements in the *indica* variety 93‐11 genome.

**Figure S6.** PCR analysis to detect co‐transformed transgenic plants.

**Figure S7.** Validation of the *mJing* insertion identified through targeted high‐throughput sequencing using PCR analysis.

**Figure S8.** Distribution and insertion preference of 297 *mJing*‐like elements identified through targeted high‐throughput sequencing.

**Figure S9.** Flow chart of the method used for targeted high‐throughput sequencing.

**Table S1.** Analysis of *mJing* excisions within the *htd1* mutant allele in the F_3_ through F_6_ generations.

**Table S2.** Detailed information on 72 *mJing*‐like elements in the *japonica* rice variety Nipponbare genome (Os‐Nipponbare‐Reference‐IRGSP‐1.0, MSU7).

**Table S3.** Characteristics of target site duplications (TSDs) of 72 *mJing*‐like elements in the *japonica* variety Nipponbare genome (Os‐Nipponbare‐Reference‐IRGSP‐1.0, MSU7).

**Table S4.** Detailed information on 79 *mJing*‐like elements in the *indica* rice variety 93‐11 genome (ASM465v1).

**Table S5.** Presence/absence polymorphism of 297 *mJing* elements in the 71 samples surveyed.

**Table S6.** Characteristics of target site duplications (TSDs) of 297 *mJing* elements identified through targeted high‐throughput sequencing.

**Table S7.** Detailed information on 297 *mJing* elements identified through targeted high‐throughput sequencing.

**Table S8.** Information on *mJing de novo* insertions sites found in nine F_5_ individuals in which the *mJing* element was excised at the *htd1* locus.

**Table S9.** Information of *mJing de novo* insertions found in the wild rice introgression lines.

**Table S10.** Oligonucleotides used in targeted high‐throughput sequencing.

**Table S11.** Enriched reads at the *mJing* insertions identified in *japonica* rice variety Do Khao (Chr12:25 499 426) and *O. rufipogon* accession IRGC89140 (Chr1:14 969 394).

**Table S12.** Primers used in this study.

## Data Availability

The GenBank accessions for nucleotide sequences of *mJing* and *Jing* identified in this study are MH727588 (https://www.ncbi.nlm.nih.gov/nuccore/MH727588) and MH727589 (https://www.ncbi.nlm.nih.gov/nuccore/MH727589), respectively. Clean data generated in targeted high‐throughput sequencing in this study have been deposited in NCBI's Short Read Archive under the accession PRJNA507518 (https://www.ncbi.nlm.nih.gov/sra/PRJNA507518).
