# Supplementary material for: Identification of an active miniature inverted‐repeat transposable element mJing in rice
Source: Plant J. 2019 Mar 1;98(4):639–53. doi: 10.1111/tpj.14260 (PMC6850418; doi:10.1111/tpj.14260)
Supplement: Supplementary file 1 — Figure S1. Phenotypes of wild‐type (WT) and htd mutant rice. [file TPJ-98-639-s001.pdf]

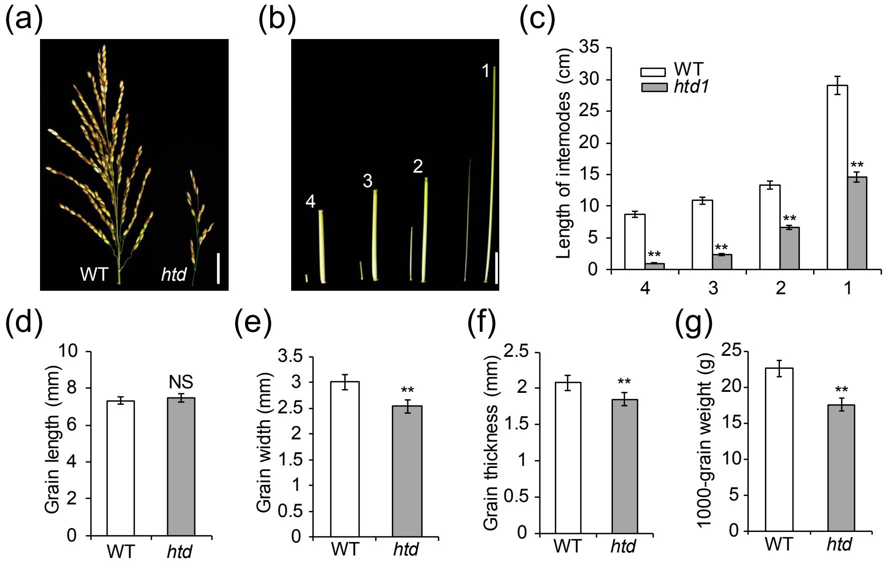

Figure S1. Phenotypes of wild type (WT) and *htd* mutant rice.

(a) Comparison of the main panicles of WT and *htd* at the harvest stage. Scale bar = 2 cm.

(b) and (c) Comparison of internode length in WT and *htd* (shown on the left and right, respectively) (b). The positions (1 to 4) from the upper to lower internode are indicated. Scale bar = 5 cm. Values are the mean ± S.D. (n = 20). Two-tailed Student's *t*-tests were performed (** $P < 0.01$).

(d)–(g) Comparison of the grain length (d), grain width (e), grain thickness (f), and 1,000-grain weight (g) of WT vs. *htd* plants. Values are the mean ± S.D. (n = 20). Two-tailed Student's *t*-tests were performed (** $P < 0.01$).
