# Supplementary material for: Identification of an active miniature inverted‐repeat transposable element mJing in rice
Source: Plant J. 2019 Mar 1;98(4):639–53. doi: 10.1111/tpj.14260 (PMC6850418; doi:10.1111/tpj.14260)
Supplement: Supplementary file 2 — Figure S2. Open reading frame (ORF) of HTD1 and deduced amino acid sequences in WT and htd. [file TPJ-98-639-s002.pdf]

```
WT   1     ATGGCAACACAAGCGATTGCACCGATGCACGCCGCCGTCGTGCACCGCCACCACGTTCTA  60
            M  A  T  Q  A  I  A  P  M  H  A  A  V  V  H  R  H  H  V  L
htd  1     ATGGCAACACAAGCGATTGCACCGATGCACGCCGCCGTCGTGCACCGCCACCACGTTCTA  60
            M  A  T  Q  A  I  A  P  M  H  A  A  V  V  H  R  H  H  V  L

WT   61    CCACCCCGCCGCTGCGTGCGCCGCCGTGGCGTCTTCGTCCGCGCCTCGGCCGCCGCCGCC  120
            P  P  R  R  C  V  R  R  R  G  V  F  V  R  A  S  A  A  A  A
htd  61    CCACCCCGCCGCTGCGTGCGCCGCCGTGGCGTCTTCGTCCGCGCCTCGGCCGCCGCCGCC  120
            P  P  R  R  C  V  R  R  R  G  V  F  V  R  A  S  A  A  A  A

WT   121   GCCGCCGCCGCCGAGACGGACACGCTGTCCGCGGCCTTCTGGGACTACAACCTCCTCTTC  180
            A  A  A  A  E  T  D  T  L  S  A  A  F  W  D  Y  N  L  L  F
htd  121   GCCGCCGCCGCCGAGACGGACACGCTGTCCGCGGCCTTCTGGGACTACAACCTCCTCTTC  180
            A  A  A  A  E  T  D  T  L  S  A  A  F  W  D  Y  N  L  L  F

WT   181   CGGTCGCAGCGCGACGAGTGCCTCGACTCCATCCCGCTCCGCGTCACCGAGGGCGCGATC  240
            R  S  Q  R  D  E  C  L  D  S  I  P  L  R  V  T  E  G  A  I
htd  181   CGGTCGCAGCGCGACGAGTGCCTCGACTCCATCCCGCTCCGCGTCACCGAGGGCGCGATC  240
            R  S  Q  R  D  E  C  L  D  S  I  P  L  R  V  T  E  G  A  I

WT   241   CCGCCCGACTTCCCGGCCGGCACCTACTACCTCGCCGGGCCGGGCATCTTCTCCGACGAC  300
            P  P  D  F  P  A  G  T  Y  Y  L  A  G  P  G  I  F  S  D  D
htd  241   CCGCCCGACTTCCCGGCCGGCACCTACTACCTCGCCGGGCCGGGCATCTTCTCCGACGAC  300
            P  P  D  F  P  A  G  T  Y  Y  L  A  G  P  G  I  F  S  D  D

WT   301   CACGGCTCCACCGTCCACCCCCTCGACGGCCACGGCTACCTCCGCTCCTTCCGCTTCCGG  360
            H  G  S  T  V  H  P  L  D  G  H  G  Y  L  R  S  F  R  F  R
htd  301   CACGGCTCCACCGTCCACCCCCTCGACGGCCACGGCTACCTCCGCTCCTTCCGCTTCCGG  360
            H  G  S  T  V  H  P  L  D  G  H  G  Y  L  R  S  F  R  F  R

WT   361   CCCGGCGACCGCACCATCCACTACTCCGCGCGGTTCGTGGAGACGGCGGCGAAGAGGGAG  420
            P  G  D  R  T  I  H  Y  S  A  R  F  V  E  T  A  A  K  R  E
htd  361   CCCGGCGACCGCACCATCCACTACTCCGCGCGGTTCGTGGAGACGGCGGCGAAGAGGGAG  420
            P  G  D  R  T  I  H  Y  S  A  R  F  V  E  T  A  A  K  R  E

WT   421   GAGAGCCGGGACGGCGCGTCGTGGCGGTTCACGCACCGGGGGCCCTTCTCCGTGCTGCAG  480
            E  S  R  D  G  A  S  W  R  F  T  H  R  G  P  F  S  V  L  Q
htd  421   GAGAGCCGGGACGGCGCGTCGTGGCGGTTCACGCACCGGGGGCCCTTCTCCGTGCTGCAG  480
            E  S  R  D  G  A  S  W  R  F  T  H  R  G  P  F  S  V  L  Q

WT   481   GGCGGGAAGAAGGTGGGCAATGTGAAGGTGATGAAGAACGTGGCCAACACCAGCGTGCTG  540
            G  G  K  K  V  G  N  V  K  V  M  K  N  V  A  N  T  S  V  L
htd  481   GGCGGGAAGAAGGTGGGCAATGTGAAGGTGATGAAGAACGTGGCCAACACCAGCGTGCTG  540
            G  G  K  K  V  G  N  V  K  V  M  K  N  V  A  N  T  S  V  L

WT   541   CGGTGGGGCGGCCGGCTGCTCTGCCTCTGGGAGGGCGGCCAGCCGTACGAGGTTGACCCC  600
            R  W  G  G  R  L  L  C  L  W  E  G  G  Q  P  Y  E  V  D  P
htd  541   CGGTGGGGCGGCCGGCTGCTCTGCCTCTGGGAGGGCGGCCAGCCGTACGAGGTTGACCCC  600
            R  W  G  G  R  L  L  C  L  W  E  G  G  Q  P  Y  E  V  D  P

WT   601   CGGACGCTCGAGACCGTCGGCCCGTTCGACCTGCTCGGCCTCGCCGCCGCCGACGACAAC  660
            R  T  L  E  T  V  G  P  F  D  L  L  G  L  A  A  A  D  D  N
htd  601   CGGACGCTCGAGACCGTCGGCCCGTTCGACCTGCTCGGCCTCGCCGCCGCCGACGACAAC  660
            R  T  L  E  T  V  G  P  F  D  L  L  G  L  A  A  A  D  D  N

WT   661   AAGGCAACGAACGCGTCTGCAGCACGACGGCCGTGGCTGCAGGAGGCCGGCCTCGACGCC  720
            K  A  T  N  A  S  A  A  R  R  P  W  L  Q  E  A  G  L  D  A
htd  661   AAGGCAACGAACGCGTCTGCAGCACGACGGCCGTGGCTGCAGGAGGCCGGCCTCGACGCC  720
            K  A  T  N  A  S  A  A  R  R  P  W  L  Q  E  A  G  L  D  A

WT   721   GCCGCGCGCCTGCTGCGCCCTGTTCTTAGCGGGGTGTTCGACATGCCGGGCAAGAGGCTG  780
            A  A  R  L  L  R  P  V  L  S  G  V  F  D  M  P  G  K  R  L
htd  721   GCCGCGCGCCTGCTGCGCCCTGTTCTTAGCGGGGTGTTCGACATGCCGGGCAAGAGGCTG  780
            A  A  R  L  L  R  P  V  L  S  G  V  F  D  M  P  G  K  R  L

WT   781   CTGGCGCACTACAAGATCGACCCGCGGCGGGGGCGTCTGCTGATGGTCGCCTGCAACGCC  840
            L  A  H  Y  K  I  D  P  R  R  G  R  L  L  M  V  A  C  N  A
htd  781   CTGGCGCACTACAAGATCGACCCGCGGCGGGGGCGTCTGCTGATGGTCGCCTGCAACGCC  840
            L  A  H  Y  K  I  D  P  R  R  G  R  L  L  M  V  A  C  N  A

WT   841   GAGGACATGCTCCTCCCGCGATCCCACTTCACTTTCTACGAGTTCGACGCCCACTTCGAC  900
            E  D  M  L  L  P  R  S  H  F  T  F  Y  E  F  D  A  H  F  D
htd  841   GAGGACATGCTCCTCCCGCGATCCCACTTCACTTTCTACGAGTTCGACGCCCACTTCGAC  900
            E  D  M  L  L  P  R  S  H  F  T  F  Y  E  F  D  A  H  F  D

WT   901   CTCGTCCAGAAGCGTGAGTTCGTCGTGCCGGACCACCTCATGATCCACGACTGGGCCTTC  960
            L  V  Q  K  R  E  F  V  V  P  D  H  L  M  I  H  D  W  A  F
htd  901   CTCGTCCAGAAGCGTGAGTTCGTCGTGCCGGACCACCTCATGATCCACGACTGGGCCTTC  960
            L  V  Q  K  R  E  F  V  V  P  D  H  L  M  I  H  D  W  A  F

WT   961   ACCGACACCCACTACATCCTCCTCGGCAACAGGATCAAGCTCGACATCCCCGGATCGCTG  1020
            T  D  T  H  Y  I  L  L  G  N  R  I  K  L  D  I  P  G  S  L
htd  961   ACCGACACCCACTACATCCTCCTCGGCAACAGGATCAAGCTCGACATCCCCGGATCGCTG  1020
            T  D  T  H  Y  I  L  L  G  N  R  I  K  L  D  I  P  G  S  L

WT   1021  CTGGCATTGACGGGCACTCACCCGATGATCGCGGCGCTGGCCGTGGACCCGAGAAGGCAG  1080
            L  A  L  T  G  T  H  P  M  I  A  A  L  A  V  D  P  R  R  Q
htd  1021  CTGGCATTGACGGGCACTCACCCGATGATCGCGGCGCTGGCCGTGGACCCGAGAAGGCAG  1080
            L  A  L  T  G  T  H  P  M  I  A  A  L  A  V  D  P  R  R  Q

WT   1081  TCGACGCCGGTGTACCTGCTTCCGCGCTCCCCGGAGACCGAGGCGGGCGGCCGCGACTGG  1140
            S  T  P  V  Y  L  L  P  R  S  P  E  T  E  A  G  G  R  D  W
htd  1081  TCGACGCCGGTGTACCTGCTTCCGCGCTCCCCGGAGACCGAGGCGGGCGGCCGCGACTGG  1140
            S  T  P  V  Y  L  L  P  R  S  P  E  T  E  A  G  G  R  D  W
```

```
WT   1141 AGCGTGCCGATCGAGGCGCCGTCGCAGATGTGGTCCGTGCACGTCGGCAACGCGTTCGAG 1200
            S  V  P  I  E  A  P  S  Q  M  W  S  V  H  V  G  N  A  F  E
htd  1141 AGCGTGCCGATCGAGGCGCCGTCGCAGATGTGGTCCGTGCACGTCGGCAACGCGTTCGAG 1200
            S  V  P  I  E  A  P  S  Q  M  W  S  V  H  V  G  N  A  F  E

WT   1201 GAGGCGAACCGCCGGGGCGGCCTCGACGTCCGGCTGCACATGTCAAGCTGCTCCTACCAG 1260
            E  A  N  R  R  G  G  L  D  V  R  L  H  M  S  S  C  S  Y  Q
htd  1201 GAGGCGAACCGCCGGGGCGGCCTCGACGTCCGGCTGCACATGTCAAGCTGCTCCTACCAG 1260
            E  A  N  R  R  G  G  L  D  V  R  L  H  M  S  S  C  S  Y  Q

WT   1261 TGGTTCCATTTCCACAGGATGTTTGGTTACAATTGGCACCACAAGAAGCTGGACCCGTCG 1320
            W  F  H  F  H  R  M  F  G  Y  N  W  H  H  K  K  L  D  P  S
htd  1261 TGGTTCCATTTCCACAGGATGTTTGGTTACAATTGGCACCACAAGAAGCTGGACCCGTCG 1320
            W  F  H  F  H  R  M  F  G  Y  N  W  H  H  K  K  L  D  P  S

WT   1321 TTCATGAACGCGGCGAAGGGAAAGGAGTGGCTGCCTCGCCTCGTTCAGGTGGCCATCGAG 1380
            F  M  N  A  A  K  G  K  E  W  L  P  R  L  V  Q  V  A  I  E
htd  1321 TTCATGAACGCGGCGAAGGGAAAGGAGTGGCTGCCTCGCCTCGTTCAGGTGGCCATCGAG 1380
            F  M  N  A  A  K  G  K  E  W  L  P  R  L  V  Q  V  A  I  E

WT   1381 CTCGACAGGACGGGAGAGTGCCGGAGGTGCTCAGTCAGGAGGCTGTCCGATCAGCACGCC 1440
            L  D  R  T  G  E  C  R  R  C  S  V  R  R  L  S  D  Q  H  A
htd  1381 CTCGACAGGACGGGAGAGTGCCGGAGGTGCTCAGTCAGGAGGCTGTCCGATCAGCACGCC 1440
            L  D  R  T  G  E  C  R  R  C  S  V  R  R  L  S  D  Q  H  A

WT   1441 AGGCCGGCGGACTTCCCGGCGATAAACCCAAGCTACGCCAACCAGAGGAACCGGTTCGTC 1500
            R  P  A  D  F  P  A  I  N  P  S  Y  A  N  Q  R  N  R  F  V
htd  1441 AGGCCGGCGGACTTCCCGGCGATAAACCCAAGCTACGCCAACCAGAGGAACCGGTTCGTC 1500
            R  P  A  D  F  P  A  I  N  P  S  Y  A  N  Q  R  N  R  F  V

WT   1501 ------------------------------------------------------------ 1504
htd  1501 tacggctgcgttcgtttggacgttttcccaccgcgctacagtaacacggaaaacggaacg 1560
            Y  G  C  V  R  L  D  V  F  P  P  R  Y  S  N  T  E  N  G  T

WT   1504 ------------------------------------------------------------ 1504
htd  1561 gttcattaacacgtgattaattaagtattagttaatttttttttcaaaaatggattaatt 1620
            V  H  *  -  -  -  -  -  -  -  -  -  -  -  -  -  -  -  -  -

WT   1504 ------------------------------------------------------------ 1504
htd  1621 tgatttttttaagcaacttttgtatagaaactttttgcaaaaaagtacaccgtgtagtag 1680
            -  -  -  -  -  -  -  -  -  -  -  -  -  -  -  -  -  -  -  -

WT   1504 ------------------------------------------------------------ 1504
htd  1681 tttgaaaagcgtgcgcgggggcaaaacactgtagcgcccgctgaaagtgctgtgccgaac 1740
            -  -  -  -  -  -  -  -  -  -  -  -  -  -  -  -  -  -  -  -

WT   1504 ------TACGCCGGCGCCGCGTCCGGCTCCCGCAGATTCCTCCCGTACTTCCCGTTCGAC 1554
                   Y  A  G  A  A  S  G  S  R  R  F  L  P  Y  F  P  F  D
htd  1741 acagccTACGCCGGCGCCGCGTCCGGCTCCCGCAGATTCCTCCCGTACTTCCCGTTCGAC 1800
            -  -  -  -  -  -  -  -  -  -  -  -  -  -  -  -  -  -  -  -

WT   1555 AGCGTGGTGAAGGTCGACGTCTCCGATGGATCGGCGCGGTGGTGGTCTACCGACGGGCGC 1614
            S  V  V  K  V  D  V  S  D  G  S  A  R  W  W  S  T  D  G  R
htd  1801 AGCGTGGTGAAGGTCGACGTCTCCGATGGATCGGCGCGGTGGTGGTCTACCGACGGGCGC 1860
            -  -  -  -  -  -  -  -  -  -  -  -  -  -  -  -  -  -  -  -

WT   1615 AAGTTCGTCGGCGAGCCGGTCTTCGTCCCGACCGGCGGCGGAGAGGATGGTGGCTATGTT 1674
            K  F  V  G  E  P  V  F  V  P  T  G  G  G  E  D  G  G  Y  V
htd  1861 AAGTTCGTCGGCGAGCCGGTCTTCGTCCCGACCGGCGGCGGAGAGGATGGTGGCTATGTT 1920
            -  -  -  -  -  -  -  -  -  -  -  -  -  -  -  -  -  -  -  -

WT   1675 CTTCTTGTAGAGTATGCAGTCTCCAAGCACAGATGCCATCTAGTGGTGCTGGATGCAAAG 1734
            L  L  V  E  Y  A  V  S  K  H  R  C  H  L  V  V  L  D  A  K
htd  1921 CTTCTTGTAGAGTATGCAGTCTCCAAGCACAGATGCCATCTAGTGGTGCTGGATGCAAAG 1980
            -  -  -  -  -  -  -  -  -  -  -  -  -  -  -  -  -  -  -  -

WT   1735 AAGATAGGGACAGAGAATGCACTTGTGGCAAAACTAGAGGTGCCAAAGAACCTCACTTTT 1794
            K  I  G  T  E  N  A  L  V  A  K  L  E  V  P  K  N  L  T  F
htd  1981 AAGATAGGGACAGAGAATGCACTTGTGGCAAAACTAGAGGTGCCAAAGAACCTCACTTTT 2040
            -  -  -  -  -  -  -  -  -  -  -  -  -  -  -  -  -  -  -  -

WT   1795 CCAATGGGATTCCATGGTTTCTGGGGAGATGAATGA 1830
            P  M  G  F  H  G  F  W  G  D  E  *
htd  2041 CCAATGGGATTCCATGGTTTCTGGGGAGATGAATGA 2076
            -  -  -  -  -  -  -  -  -  -  -  -
```

Figure S2. The open reading frame (ORF) of *HTD1* and deduced amino acid sequences in WT and *htd*. Black uppercase letters represent the ORF sequences. Red lowercase letters represent the *mJing* insertion. Blue uppercase letters represent amino acid residues. Asterisks indicate the stop codon.
