# Supplementary material for: Identification of an active miniature inverted‐repeat transposable element mJing in rice
Source: Plant J. 2019 Mar 1;98(4):639–53. doi: 10.1111/tpj.14260 (PMC6850418; doi:10.1111/tpj.14260)
Supplement: Supplementary file 3 — Figure S3. Transposition of mJing in a high‐tillering dwarf population. [file TPJ-98-639-s003.pdf]

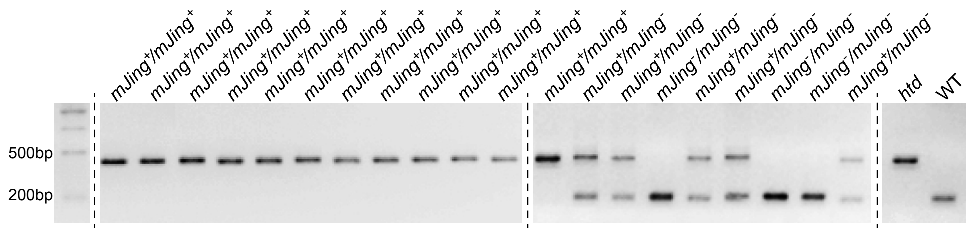

Figure S3. The transposition of *mJing* in a high-tillering dwarf population.

Detection of *mJing* excision in high-tillering, dwarf F_4 individuals through PCR using primer set ID-6. *mJing*$^+$ and *mJing*$^-$ represent the insertion and excision of *mJing*, respectively.
