# Supplementary material for: Identification of an active miniature inverted‐repeat transposable element mJing in rice
Source: Plant J. 2019 Mar 1;98(4):639–53. doi: 10.1111/tpj.14260 (PMC6850418; doi:10.1111/tpj.14260)
Supplement: Supplementary file 4 — Figure S4. Changes in amino acid sequence encoded by the htd1 alleles and phenotypes of F4 individuals that have the mJing +/mJing − or mJing −/mJing − genotype. [file TPJ-98-639-s004.pdf]

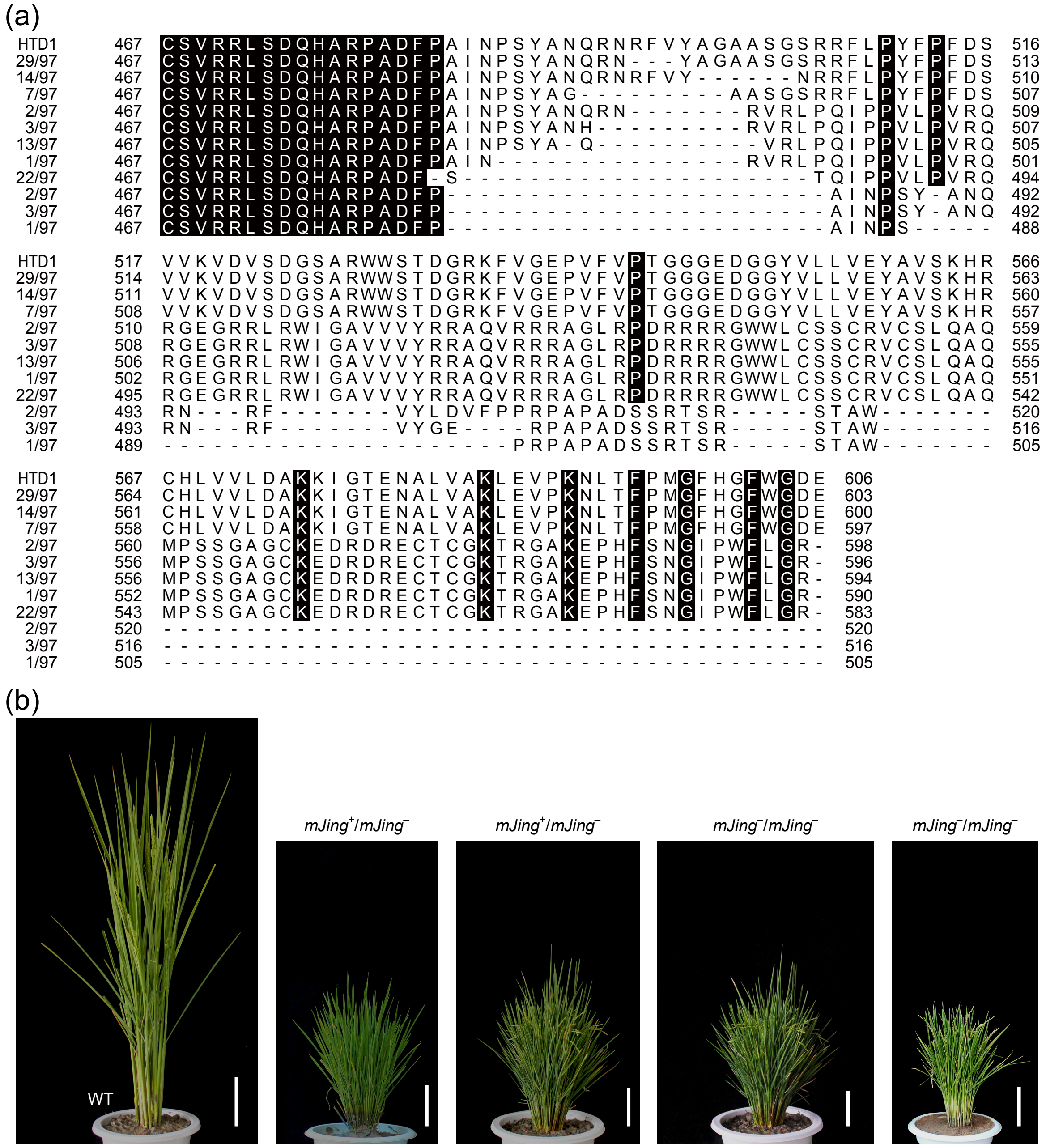

Figure S4. The changes of amino acid sequence encoded by the *htd1* alleles and phenotypes of the F_4 individuals with *mJing*$^+$/*mJing*$^-$ or *mJing*$^-$/*mJing*$^-$ genotypes.

(a) The alignment of partial amino acid sequences encoded by the *htd1* alleles in which the *mJing* MITE was imprecisely excised. Black boxes represent the identical amino acid residue to wild type.

(b) Phenotypes of the wild type (WT) and the F_4 individuals with *mJing*$^+$/*mJing*$^-$ or *mJing*$^-$/*mJing*$^-$ genotypes. *mJing*$^+$ and *mJing*$^-$ represent the insertion and excision of *mJing* at the *htd1* locus, respectively. Scale bar = 10 cm.
