# Supplementary material for: Identification of an active miniature inverted‐repeat transposable element mJing in rice
Source: Plant J. 2019 Mar 1;98(4):639–53. doi: 10.1111/tpj.14260 (PMC6850418; doi:10.1111/tpj.14260)
Supplement: Supplementary file 5 — Figure S5. Consensus sequences of target site duplications (TSDs) and terminal inverted repeats (TIRs) of 79 mJing‐like elements in the indica variety 93‐11 genome. [file TPJ-98-639-s005.pdf]

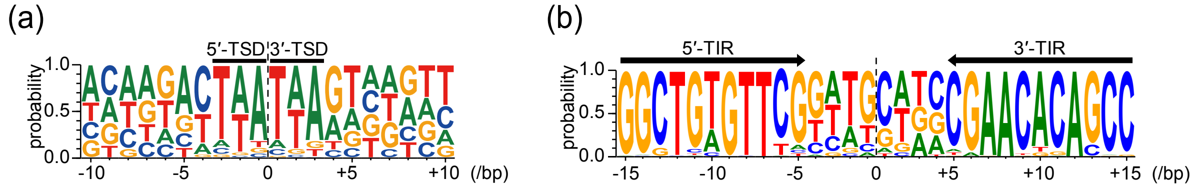

Figure S5. Consensus sequences of target site duplications (TSDs) (a) and terminal inverted repeats (TIRs) (b) of the 79 *mJing*-like elements in the *indica* variety 93-11 genome. The letter size indicates the frequency of the corresponding nucleotide. Black lines and arrows above the letters indicate the TSDs and TIRs in (a) and (b), respectively.
