# Supplementary material for: Identification of an active miniature inverted‐repeat transposable element mJing in rice
Source: Plant J. 2019 Mar 1;98(4):639–53. doi: 10.1111/tpj.14260 (PMC6850418; doi:10.1111/tpj.14260)
Supplement: Supplementary file 6 — Figure S6. PCR analysis to detect co‐transformed transgenic plants. [file TPJ-98-639-s006.pdf]

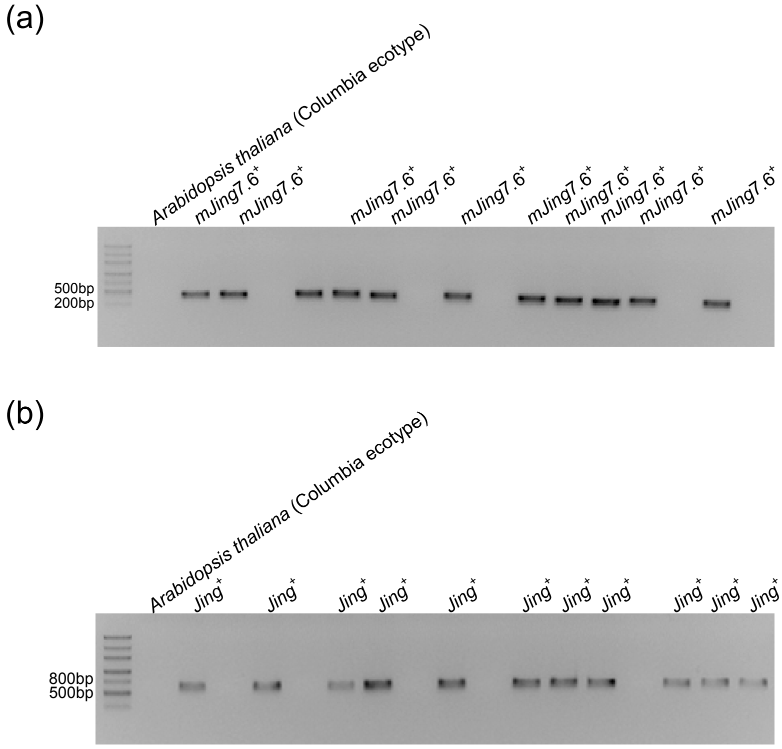


**Figure S6.** PCR analysis to detect co-transformed transgenic plants.

(a) Detection of co-transformed transgenic plants carrying *mJing*7.6 by PCR analysis using primer set D_p*mJing*7.6-2. *mJing*7.6$^{+}$ represents a transgenic plant containing an entire *mJing*7.6 element.

(b) Detection of co-transformed transgenic plants carrying *Jing* by PCR analysis using primer set D_p*35S::Jing*. *Jing*$^{+}$ represents a transgenic plant containing *Jing* element.
