# Supplementary material for: Identification of an active miniature inverted‐repeat transposable element mJing in rice
Source: Plant J. 2019 Mar 1;98(4):639–53. doi: 10.1111/tpj.14260 (PMC6850418; doi:10.1111/tpj.14260)
Supplement: Supplementary file 7 — Figure S7. Validation of the mJing insertion identified through targeted high‐throughput sequencing using PCR analysis. [file TPJ-98-639-s007.pdf]

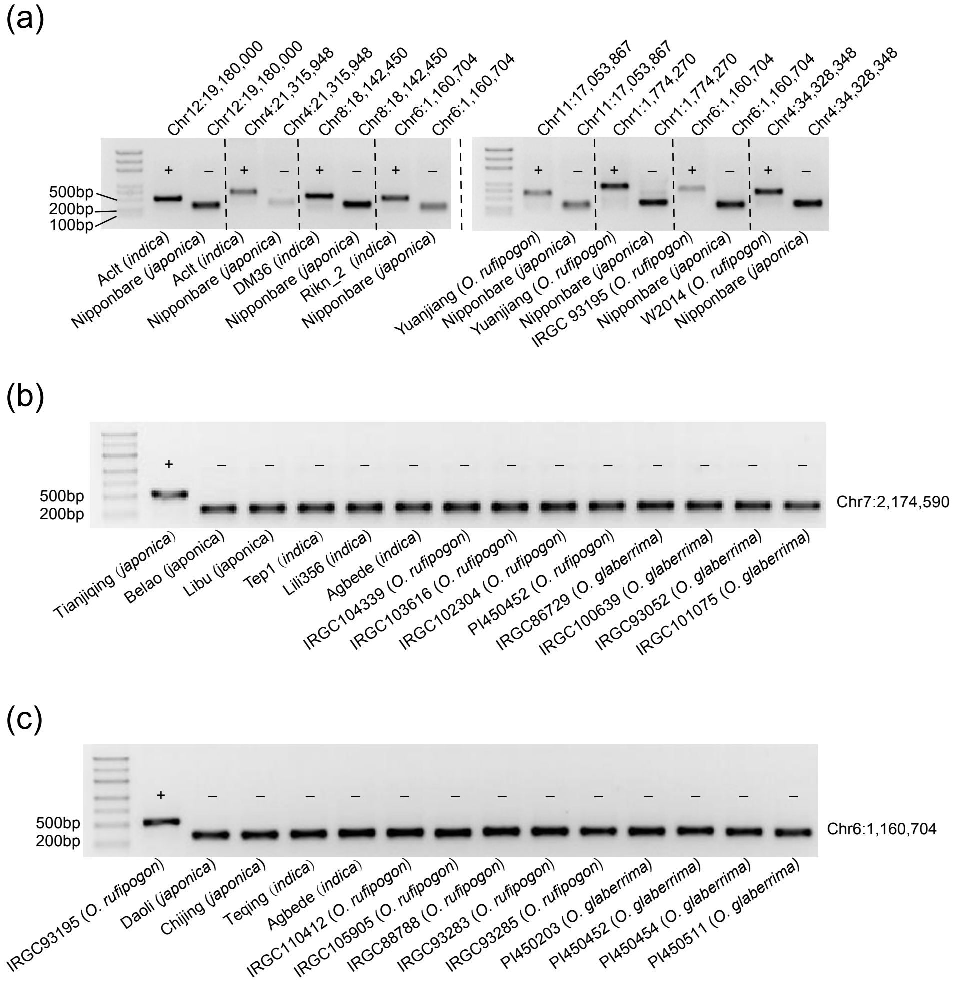

Figure S7. Validation of the *mJing* insertion identified through targeted high-throughput sequencing using PCR analysis.

(a) Validation of the *mJing* insertion using locus-specific PCR analysis. Plus and minus represent the presence and absence of the *mJing* insertion, respectively. *Japonica* rice variety Nipponbare was used as the control.

(b) and (c) Validation of the unique *mJing* insertions in *japonica* variety Tianjiqing (Chr7:21,745,907) (b) and *O. rufipogon* accession IRGC93195 (Chr6:1,160,704) (c). Plus and minus represent the presence and absence of the *mJing* insertion, respectively.
