# Supplementary material for: Identification of an active miniature inverted‐repeat transposable element mJing in rice
Source: Plant J. 2019 Mar 1;98(4):639–53. doi: 10.1111/tpj.14260 (PMC6850418; doi:10.1111/tpj.14260)
Supplement: Supplementary file 8 — Figure S8. Distribution and insertion preference of 297 mJing‐like elements identified through targeted high‐throughput sequencing. [file TPJ-98-639-s008.pdf]

(a)

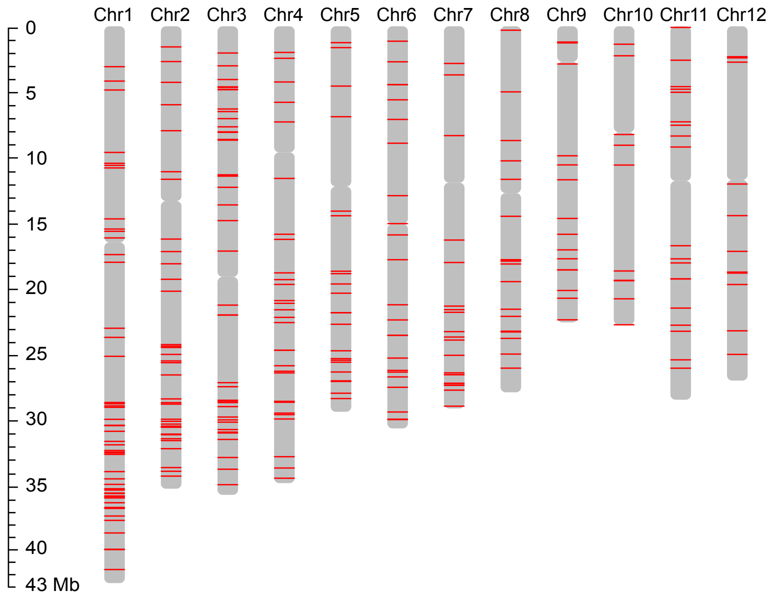

(b)

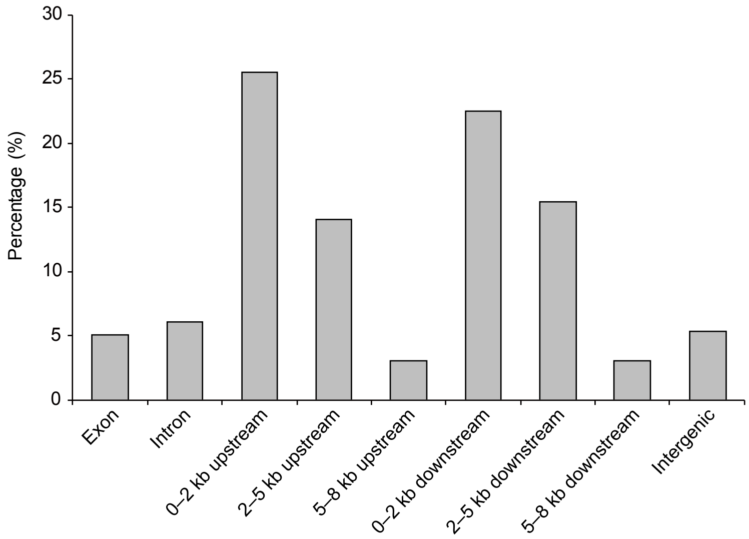

Figure S8. Distribution (a) and insertion preference (b) of the 297 *mJing*-like elements identified through targeted high-throughput sequencing.
