# Supplementary figures and images for: Identification of an active miniature inverted‐repeat transposable element mJing in rice
Source: Plant J. 2019 Mar 1;98(4):639–53. doi: 10.1111/tpj.14260 (PMC6850418; doi:10.1111/tpj.14260)

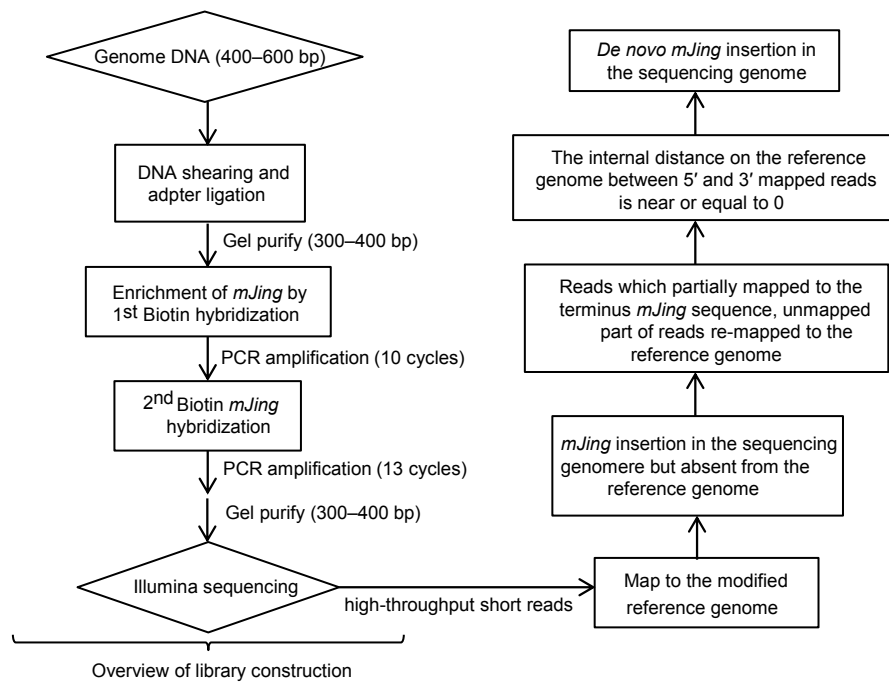

**Figure S9.** Flowchart of the method used for targeted high-throughput sequencing.

Supplement: Supplementary file 9 — Figure S9. Flow chart of the method used for targeted high‐throughput sequencing. [file TPJ-98-639-s009.pdf]
