# Supplementary material for: Identification of an active miniature inverted‐repeat transposable element mJing in rice
Source: Plant J. 2019 Mar 1;98(4):639–53. doi: 10.1111/tpj.14260 (PMC6850418; doi:10.1111/tpj.14260)
Supplement: Supplementary file 22 [file TPJ-98-639-s022.docx]

**Figure S1.** Phenotypes of wild type (WT) and *htd* mutant rice.

(a) Comparison of the main panicles of WT and *htd* at the harvest stage. Scale bar = 2 cm.

(b) and (c) Comparison of internode length in WT and *htd* (shown on the left and right, respectively) (b)*.* The positions (1 to 4) from the upper to lower internode are indicated. Scale bar = 5 cm. Values are the mean ± S.D. (n = 20). Two-tailed Student’s *t*-tests were performed (** *P* < 0.01).

(d)–(g) Comparison of the grain length (d), grain width (e), grain thickness (f), and 1,000-grain weight (g) of WT vs. *htd* plants. Values are the mean ± S.D. (n = 20). Two-tailed Student’s *t*-tests were performed (** *P* < 0.01).

**Figure S2.** The open reading frame (ORF) of *HTD1* and deduced amino acid sequences in WT and *htd*.

Black uppercase letters represent the ORF sequences. Red lowercase letters represent the *mJing* insertion. Blue uppercase letters represent amino acid residues. Asterisks indicate the stop codon.

**Figure S3.** The transposition of *mJing* in a high-tillering dwarf population.

Detection of *mJing* excision in high-tillering, dwarf F_4_ individuals through PCR using primer set ID-6. *mJing*^+^ and *mJing*^–^ represent the insertion and excision of *mJing*, respectively.

**Figure S4.** The changes of amino acid sequence encoded by the *htd1* alleles and phenotypes of the F_4_ individuals with *mJing*^+^/*mJing*^–^ or *mJing*^–^/*mJing*^–^ genotypes.

(a) The alignment of partial amino acid sequences encoded by the *htd1* alleles in which the *mJing* MITE was imprecisely excised. Black boxes represent the identical amino acid residue to wild type.

(b) Phenotypes of the wild type (WT) and the F_4_ individuals with *mJing*^+^/*mJing*^–^ or *mJing*^–^/*mJing*^–^ genotypes. *mJing*^+^ and *mJing*^–^ represent the insertion and excision of *mJing* at the *htd1* locus, respectively. Scale bar = 10 cm.

**Figure S5.** Consensus sequences of target site duplications (TSDs) (a) and terminal inverted repeats (TIRs) (b) of the 79 *mJing*-like elements in the *indica* variety 93-11 genome. The letter size indicates the frequency of the corresponding nucleotide. Black lines and arrows above the letters indicate the TSDs and TIRs in (a) and (b), respectively.

**Figure S6.** PCR analysis to detect co-transformed transgenic plants.

(a) Detection of co-transformed transgenic plants carrying *mJing*7.6 by PCR analysis using primer set D_p*mJing*7.6-2. *mJing*7.6^+^ represents a transgenic plant containing an entire *mJing*7.6 element.

(b) Detection of co-transformed transgenic plants carrying *Jing* by PCR analysis using primer set D_p*35S::Jing*. *Jing*^+^ represents a transgenic plant containing *Jing* element.

**Figure S7.** Validation of the *mJing* insertion identified through targeted high-throughput sequencing using PCR analysis.

(a) Validation of the *mJing* insertion using locus-specific PCR analysis. Plus and minus represent the presence and absence of the *mJing* insertion, respectively. *Japonica* rice variety Nipponbare was used as the control.

(b) and (c) Validation of the unique *mJing* insertions in *japonica* variety Tianjiqing (Chr7:21,745,907) (b) and *O. rufipogon* accession IRGC93195 (Chr6:1,160,704) (c). Plus and minus represent the presence and absence of the *mJing* insertion, respectively.

**Figure S8.** Distribution (a) and insertion preference (b) of the 297 *mJing*-like elements identified through targeted high-throughput sequencing.

**Figure S9.** Flowchart of the method used for targeted high-throughput sequencing.
